# Effects of Delayed Radical Prostatectomy and Active Surveillance on Localised Prostate Cancer—A Systematic Review and Meta-Analysis

**DOI:** 10.3390/cancers13133274

**Published:** 2021-06-30

**Authors:** Vinson Wai-Shun Chan, Wei Shen Tan, Aqua Asif, Alexander Ng, Olayinka Gbolahan, Eoin Dinneen, Wilson To, Hassan Kadhim, Melissa Premchand, Oliver Burton, Jasmine Sze-Ern Koe, Nicole Wang, Jeffrey J. Leow, Gianluca Giannarini, Nikhil Vasdev, Shahrokh F. Shariat, Dmitry Enikeev, Chi Fai Ng, Jeremy Yuen-Chun Teoh

**Affiliations:** 1 School of Medicine, Faculty of Medicine and Health, University of Leeds, Leeds LS2 9JT, UK; um19jsek@leeds.ac.uk; 2 S.H. Ho Urology Centre, Department of Surgery, Prince of Wales Hospital, The Chinese University of Hong Kong, Hong Kong, China; ngcf@surgery.cuhk.edu.hk; 3 Division of Surgery and Interventional Science, University College London, London W1W 7TS, UK; wei.tan@ucl.ac.uk (W.S.T.); aqua.asif@ucl.ac.uk (A.A.); eoin.dinneen@nhs.net (E.D.); 4 Department of Urology, Royal Free Hospital, London NW3 2QG, UK; olayinka.gbolahan@nhs.net (O.G.); wilson.to2@nhs.net (W.T.); 5 Leicester Medical School, University of Leicester, Leicester LE1 7RH, UK; 6 UCL Medical School, University College London, London WC1E 6DE, UK; alexander.ng.16@ucl.ac.uk (A.N.); nicole.wang.17@ucl.ac.uk (N.W.); 7 Department of Urology, Ipswich Hospital, Ipswich IP4 5PD, UK; hassankadhim@nhs.net; 8 Department of Urology, Northwick Park Hospital, London HA1 3UJ, UK; melissa.premchand@nhs.net; 9 Warwick Medical School, University of Warwick, Coventry CV4 7AL, UK; ollieburton@doctors.org.uk; 10 Department of Urology, Tan Tock Seng Hospital, Singapore 308433, Singapore; jeffrey.leow@mail.harvard.edu; 11 Urology Unit, Santa Maria della Misericordia University Hospital, 33100 Udine, Italy; gianluca.giannarini@asufc.sanita.fvg.it; 12 Department of Urology, Hertfordshire and Bedfordshire Urological Cancer Centre, Lister Hospital Stevenage, School of Medicine and Life Sciences, University of Hertfordshire, Hatfield AL10 9AB, UK; nikhil.vasdev@nhs.net; 13 Department of Urology, Medical University of Vienna, 1090 Vienna, Austria; shahrokh.shariat@meduniwien.ac.at; 14 Department of Urology, Comarcal Hospital, 17300 Monforte, Spain; 15 Institute for Urology and Reproductive Health, Sechenov University, 119991 Moscow, Russia; enikeev_d_v@staff.sechenov.ru; 16 Department of Urology, Weill Cornell Medical College, New York, NY 10065, USA; 17 Department of Urology, University of Texas Southwestern, Dallas, TX 75390, USA; 18 Department of Urology, Charles University, 116 36 Prague, Czech Republic

**Keywords:** COVID-19, prostate cancer, radical prostatectomy, treatment delay, surgical waiting time, active surveillance

## Abstract

**Simple Summary:**

We reviewed the evidence available for postponing or delaying cancer surgery for localised prostate cancer. Watchful waiting is an acceptable option in low-risk patients. Evidence is uncertain in postponing surgery, but conservative estimates suggest delays of over 5 months, 4 months, and 30 days for low-, intermediate-, and high-risk patients, respectively, can lead to worse survival outcomes. Neoadjuvant therapy can shrink the tumours prior to surgery and can be a useful adjunct in delaying surgery for, at the most, 3 months.

**Abstract:**

External factors, such as the coronavirus disease 2019 (COVID-19), can lead to cancellations and backlogs of cancer surgeries. The effects of these delays are unclear. This study summarised the evidence surrounding expectant management, delay radical prostatectomy (RP), and neoadjuvant hormone therapy (NHT) compared to immediate RP. MEDLINE and EMBASE was searched for randomised controlled trials (RCTs) and non-randomised controlled studies pertaining to the review question. Risks of biases (RoB) were evaluated using the RoB 2.0 tool and the Newcastle–Ottawa Scale. A total of 57 studies were included. Meta-analysis of four RCTs found overall survival and cancer-specific survival were significantly worsened amongst intermediate-risk patients undergoing active monitoring, observation, or watchful waiting but not in low- and high-risk patients. Evidence from 33 observational studies comparing delayed RP and immediate RP is contradictory. However, conservative estimates of delays over 5 months, 4 months, and 30 days for low-risk, intermediate-risk, and high-risk patients, respectively, have been associated with significantly worse pathological and oncological outcomes in individual studies. In 11 RCTs, a 3-month course of NHT has been shown to improve pathological outcomes in most patients, but its effect on oncological outcomes is apparently limited.

## 1. Introduction

Patients diagnosed with prostate cancer (PCa) can expect disparate times to surgical treatment based on the risk level of the detected cancer, results of biopsy, threat of metastasis, surgical waiting times, and more recently, external factors such as the coronavirus disease 2019 (COVID-19) pandemic. Over the fears of patient-to-healthcare transmission through aerosol or faecal matters during patient care, urological services have been severely impacted worldwide [1,2,3] during the pandemic. Furthermore, 30-day mortality of patients undergoing surgery with confirmed severe acute respiratory syndrome coronavirus 2 (SARS-CoV-2) infection at the time of surgery is as high as 24% [4], which further led to a predicted 2.3 million cancer surgeries being cancelled or delayed during the pandemic.

During the COVID-19 pandemic, the European Association of Urology (EAU) categorised PCa into low-, intermediate-, and high-priority based on expected clinical harm timelines (6+ months for low-priority, 3+ months for intermediate-priority, and <6 weeks for high-priority) and delays as a result of the COVID-19 pandemic should not exceed the proposed expected clinical harm timelines [5]. It is also recommended that patients undergoing active surveillance (AS) for low-risk PCa can have postponed prostate specific antigen (PSA) screening, confirmatory re-biopsies and digital rectal examinations (DREs). Active treatment can be deferred for 6–12 months. Similarly, EAU recommends that radical prostatectomy (RP) can be postponed for both intermediate-risk and high-risk patients within a safe time limit; however, neo-adjuvant hormone therapy (NHT) is not recommended in intermediate-risk patients but can be used to ease patient anxiety in high-risk cases [5].

Pandemic aside, delaying prostatectomies allows for flexibility of healthcare resources. For adequate resource allocation and patient treatment, evaluating the effects of delaying prostatectomies based on risk category is therefore necessary. Understanding the impact of prostatectomy delay with respect to the risk level of an individual presentation of PCa allows personalised care and more reserved treatments for those at low- or intermediate-risk and better prioritisation of resources for high-risk cases. 

While treatment delays or expectant management of months or years for men with low-risk PCa have not been suggested to negatively impact oncological or pathological outcomes, non-low-risk PCa present greater challenges as the risks of delaying RP in these patients are not well established [6,7,8,9]. In these patients, prostatectomy delay may suggest higher risk of oncological progression [10]. NHT has been shown to improve pathological outcomes in locally advanced PCa patients, but has not been extensively investigated to delay treatment—it may be utilised during the pandemic [11].

This study aims to evaluate the current literature for evidence surrounding the delay of RP for patients with different risks of PCa to inform the prioritisation of PCa treatments both within and outside the context of pandemics and the clearing of cases backlogged.

## 2. Materials and Methods

This review was performed according to the Preferred Reporting Items for Systematic Reviews and Meta-analyses (PRISMA) statement [12].

### 2.1. Literature Search

A comprehensive literature search was performed using medical subject headings (MeSH terms) and keywords on PubMed/MEDLINE, Cochrane Central Register of Controlled Trials (CENTRAL), and Cochrane Database of Systematic Reviews on the 16 May 2020 according to the following search strategy: “(delay OR deferral OR deferred OR interval OR (neoadjuvant AND (hormone therapy OR complete androgen blockade OR androgen ablation OR hormone treatment OR combined androgen blockade))) AND prostate cancer AND (surgery OR prostatectomy OR radical prostatectomy).” Additional articles were also sought from the reference lists of the included studies.

### 2.2. Selection Criteria

The selection criteria of this review are as follows: 

Patients: Men with low-, intermediate- or high-risk PCa.

Intervention: Any delay to RP, including surgical waiting time and NHT; or expectant management such as AS, active monitoring, watchful waiting, or observation as defined by the study.

Comparator: Immediate RP from histological diagnosis as defined by the study.

Outcomes: Oncological and pathological outcomes (outlined in Section 2.4).

Study Type: Randomised controlled trials and observational comparative studies. Non-English studies, animal studies, studies of female patients, and studies without full text were excluded. Systematic reviews, meta-analyses, letters to the editors, editorials, and single arm studies were excluded after full-text screening. 

### 2.3. Screening and Data Extraction

All retrieved records were initially screened by title and abstract against the selection criteria independently by 8 pairs of co-authors. A second screen was then performed (JJL, WST, JT) to ensure consistency. Finally, eligible articles were screened by full text using the same manner. Data extraction was then performed independently by six authors (OG, ED, WT, HK, MP) independently with discrepancy resolved by a 3rd author (WST). A second extraction was performed by six authors (VC, AA, AN, JK, OB, NW) to ensure consistency. Data on the studies (first author, year, centre, country, study design), participant demographics and oncologic characteristics, treatment characteristics, and outcomes and results were extracted using a piloted, standardised data entry form. 

### 2.4. Data Synthesis and Statistical Analysis

The primary outcome of the study is cancer-specific survival (CSS). Secondary outcomes include (biochemical) progression-free survival ([BC]PFS), overall survival (OS), (biochemical), and recurrence-free survival ([BC]RFS). Pathological outcomes such as positive surgical margin (PSM), organ confinement (OC), positive lymph nodes (PLN), seminal vesical invasion (SVI), and pathological up/down staging are also evaluated. 

As risk levelsof patients included and the time-to-event measurements for most retrospective studies are expected to differ, most of the outcomes originating from retrospective studies are synthesised qualitatively. In the event of three or more randomised studies reporting the same outcome under similar definition and time-to-event calculations meta-analysis will be performed. Survival outcomes were meta-analysed and presented as Hazard Ratios (HRs) and 95% Confidence Intervals (95% CI). Methods validated and outlined by Tierney et al. [13] were used to estimate HRs when they are not reported, as recommended by the Cochrane Collaboration [14]. Pathological outcomes are treated as dichotomous outcomes and reported using Risk Ratios (RR) and 95% CI. The random effects model (RE) was used for meta-analysis, sensitivity analyses were performed using the fixed effects model (FE). The I^2^ value was used to identify heterogeneity. A I^2^ value of 30–60%, 50–90%, and 75–100% corresponds to suggest moderate, substantial, and considerable heterogeneity, respectively [14]. Significance of heterogeneity is defined as a *p*-value < 0.10 by Cochran’s Q test.

Risks of biases of observational studies were assessed using the Newcastle Ottawa Scale (NOS) [15]. RCTs were assessed using the RoB 2.0 tool [16]. The respective risks of biases summaries are generated using the Robvis tool [17].

## 3. Results

### 3.1. Literature Search Results

**Figure 1:**
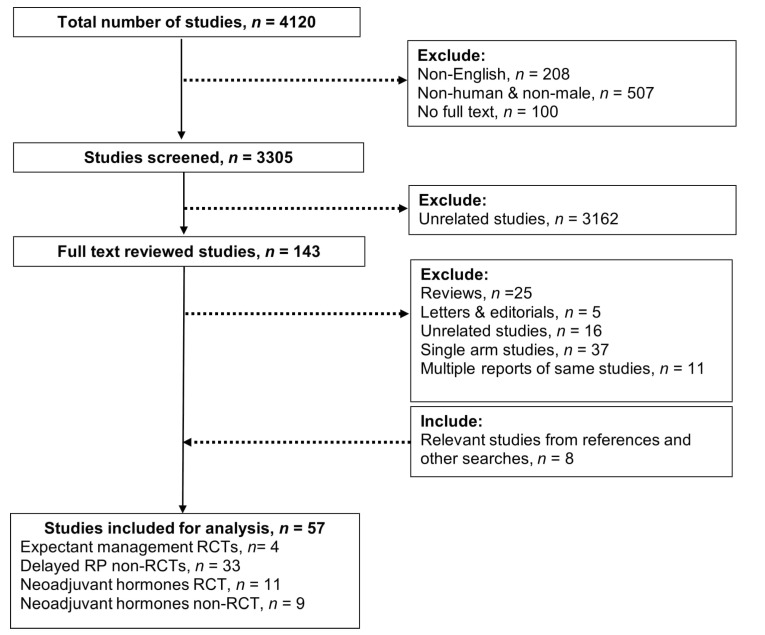
PRISMA Flow Chart.

The PRISMA flow chart is presented in Figure 1. A total of 4120 records were retrieved. After excluding unrelated studies during initial screening, 143 potential eligible articles are included for full text review. Finally, a total of 57 studies were included for this review. Of the 57 studies, 4 and 11 RCTs were included to evaluate expectant managements and neoadjuvant therapies quantitatively, respectively. Of the remaining 43 non-randomised studies, 33 reported the effect of delayed RP and 9 reported the effect of NHTs. 

### 3.2. Expectant Management

A total of four trials [6,7,8,9] have been identified comparing expectant management, active surveillance or watchful waiting with immediate RP. The study details and the risk of bias assessments of the four studies are described in Supplementary Table S1 and Supplementary Figure S1, respectively. 

#### 3.2.1. Outcomes in Mixed Risks Patients

**Figure 2:**
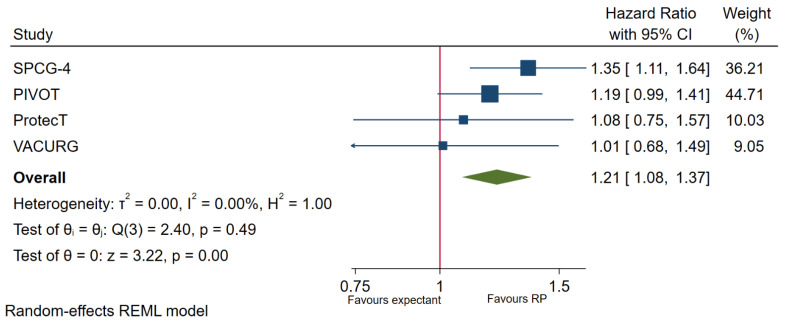
Overall survival in expectant management versus immediate RP.

**Figure 3:**
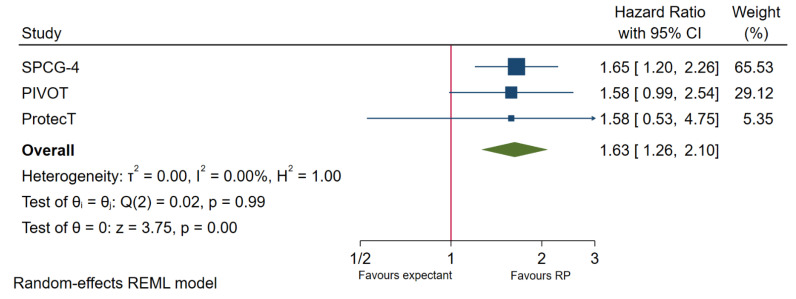
Cancer-specific survival in expectant management versus immediate RP.

In mixed risks (mixed low-, intermediate- and high-risk) patients, four trials reported OS in patients undergoing the two interventions. Overall survival was found to be significantly worse in patients not undergoing immediate RP (HR 1.21, 95% CI 1.08–1.37, *p* < 0.01; (Figure 2). While no significant heterogeneity was observed, the SPCG-4 trial population is noted to have a significantly higher proportion of T2 patients (72%), suggesting a higher risk population when compared to the other three trials. After removing the SPCG-4 from meta-analysis, expectant managements no longer have inferior OS when compared to immediate RP (HR 1.14, 95% CI 0.99–1.32, *p* = 0.08; Supplementary Figure S2). Similarly, amongst the SPCG-4, PIVOT and ProtecT trial, CSS is significantly worse in patients undergoing expectant management when compared to those undergoing immediate RP (HR 1.63, 95% CI 1.26–2.10, *p* < 0.001; Figure 3). When removing the higher risk patients in the SPCG-4 trial, CSS amongst patients undergoing expectant management is still significantly worse compared to those undergoing immediate RP (HR 1.03, 95% CI 1.03–2.44, *p* = 0.04; Supplementary Figure S3). PFS was also found to be significantly worse in patients undergoing expectant management compared to those undergoing immediate RP (HR 1.84, 95% CI 1.34–2.52, *p* < 0.001; Supplementary Figure S4). 

#### 3.2.2. Outcomes Stratified by Risks

Only two trials stratified patient outcomes into low-risk, intermediate-risk and high risk. The different classifications of risks are reported in Supplementary Table S1. In low-risk patients, OS is similar between those undergoing expectant management and immediate RP (HR 1.31, 95% CI 0.80–2.16, *p* = 0.29; Supplementary Figure S5a), despite the SPCG-4 trial having significantly more T2 patients. Similarly, CSS is similar in the two group of low-risk patients undergoing expectant management or immediate RP (HR 1.31, 95% CI 0.73–2.36, *p* = 0.36; Supplementary Figure S6a). However, PFS is significantly worse in patients undergoing expectant management compared to immediate RP (HR 2.11, 95% CI 1.29–3.45, *p* < 0.01; Supplementary Figure S7a). In intermediate-risk patients, however, OS is significantly worse in patients undergoing expectant management when compared to immediate RP (HR 1.54, 95% CI 1.21–1.98, *p* < 0.01; Supplementary Figure S5b). Similarly, CSS is also significantly worse in those undergoing expectant management (HR 1.51, 95% CI 1.65–3.82, *p* < 0.01; Supplementary Figure S6b). PFS is also significantly worse in these patients (HR 2.11, 95% CI 1.45–3.07, *p* < 0.01; Supplementary Figure S7b). In high-risk patients, both OS and CSS are similar in the two groups of patients (HR 1.23, 95% CI 0.95–1.59, *p* = 0.11; HR 1.18, 95% CI 0.77–1.81, *p* = 0.45; Supplementary Figures S5c and S6c). PFS is significantly worse in those undergoing expectant management comparted to immediate RP (HR 1.47, 95% CI 0.75–2.90, *p* < 0.01; Supplementary Figure S7c).

### 3.3. Observational Studies on Delay of RP

A total of 33 observational studies were included. The study characteristics, different definitions of risk classifications, and major outcomes and risk of bias are outlined in Supplementary Tables S2 and S3. 

#### 3.3.1. Low Risk Patients

A total of 21 studies [10,18,19,20,21,22,23,24,25,26,27,28,29,30,31,32,33,34,35,36,37] reported outcomes of delayed RP compared to immediate RP in low-risk patients. Amongst low-risk patients, evidence supporting the delay of RP are contradictory. Some studies have suggested no effect of any delay on both pathological and oncological outcomes [19,22,24,32,33,35,36]. However, it was also suggested that a delay of over 5-months may cause significantly worsened pathological and survival outcomes such as PSA failure-free survival and recurrence free survival [10,21,23,25,27,28,31,36,37]. This could be the result of the heterogeneity of investigated periods of delay from biopsy or diagnosis, ranging from 3-months to 2-years with various study designs and delay time cut off intervals.

#### 3.3.2. Intermediate-Risk Patients

A total of 11 studies [10,18,19,20,21,24,26,29,30,34,36] reported outcomes of delayed RP compared to immediate RP in intermediate-risk patients. Delaying surgery for intermediate-risk patients generally leads to significantly worse oncological and pathological outcomes. For oncological outcomes, the reported minimum delay time to cause significantly worsened BCRFS is 6-months or more [10,18,20,36]. However, a delay of four months or more was shown to lead to significantly worse pathological outcomes [10,19,27,36]. There is also significant heterogeneity of study designs and delay time cut off intervals. The investigated period of delay from biopsy or diagnosis ranged from 3 months to 9 months.

#### 3.3.3. High-Risk Patients

A total of 13 studies [10,18,19,20,21,24,26,29,30,34,38,39,40,41] reported outcomes of delayed RP compared to immediate RP in high-risk patients. A delay of 30 days or more has generally found to be associated with worsened BCRFS [10,21,24,34]. However, multiple studies have also found a delay of up to 180 days not to be associated with worsened BCRFS [19,38,39,40,41]. A delay of 30 days or more is also associated with worsened pathological outcomes [21] in one study. Some studies have suggested no effect of delay on pathological outcomes [19,22,40]. There is also a significant heterogeneity of study designs and delay time cut-off intervals. The investigated period of delay from biopsy or diagnosis ranged from 1 month to 9 months. 

### 3.4. Use of Neoadjuvant Hormone Replacement Therapy

**Figure 4:**
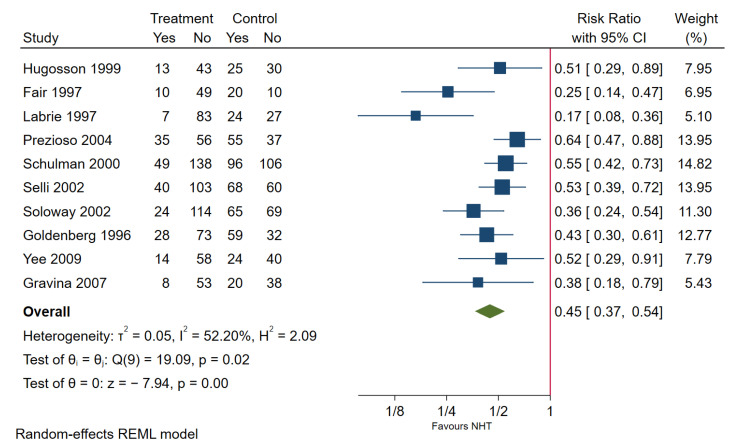
Positive surgical margin in patients undergoing 3 months NHT followed by RP or RP only.

**Figure 5:**
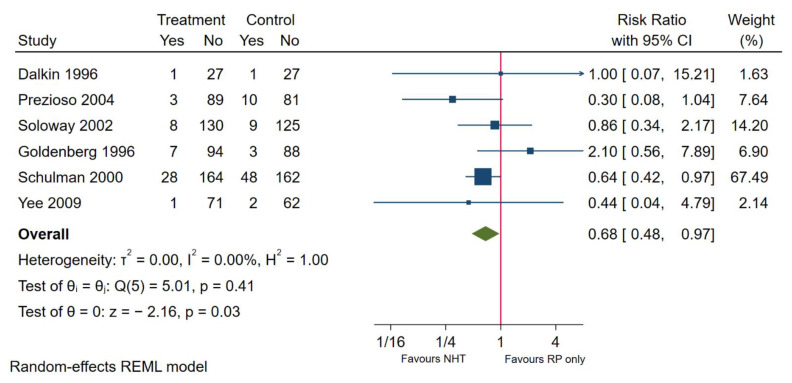
Lymph node involvement in patients undergoing 3 months NHT followed by RP or RP only.

**Figure 6:**
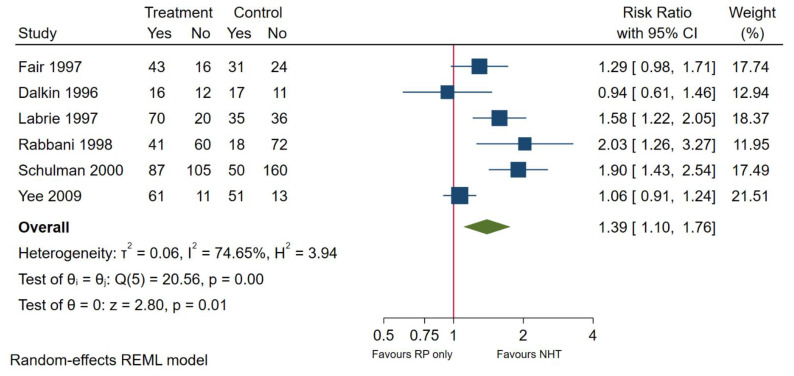
Organ confinement in patients undergoing 3 months NHT followed by RP or RP only.

A total of 21 articles [42,43,44,45,46,47,48,49,50,51,52,53,54,55,56,57,58,59,60,61,62] reported 11 RCTs comparing the use of NHTs with delayed RP and immediate RP. All studies reported 3-months courses of NHTs, one study [57] reported 6-month courses of NHT. The full treatment protocols, outcomes, and risk of biases are described in Supplementary Table S4 and Supplementary Figure S7. No significant difference between PSA failure-free survival, overall deaths, and cancer-specific deaths were found between the two groups. Patients undergoing NHT were found to have significantly lower rates of PSM (Figure 4), lymph node involvement (Figure 5), and pathological upstaging at the time of RP. Patients undergoing NHT also have significantly higher rates of organ confinement (Figure 6), and pathological down-staging at the time of RP. No differences were found between the two groups for the rates of seminal vesicle involvement. Results of meta-analyses of all outcomes after 3 months of NHT are reported in Table 1 and Supplementary Figures S8–S13. One study reported PSM after 6 months of NHT followed by RP, PSM rate was found to be significantly lower in those receiving NHT compared to those who did not [57].

Amongst the 9 non-randomised studies [63,64,65,66,67,68,69,70,71], similar results were found for both oncological and pathological outcomes in most studies. The characteristics, outcomes, and risk of biases of these studies are outlined in Supplementary Tables S5 and S6.

## 4. Discussion

To our knowledge, this is the first study to have systematically summarised the available evidence to inform the appropriate delay period to avoid worsened oncological outcomes and potential consequences in delaying RP.

In the four landmark trials [6,7,8,9] comparing active monitoring, watchful waiting, or observation to immediate RP, all but SPCG-4 found similar OS and CSS in patients undergoing expectant management and immediate RP. In contrast, the SPCG-4 trial found significantly worse OS and CSS in the watchful waiting arm compared to immediate RP treatment. The reason could be two-fold. Firstly, the SPCG-4 was conducted in a pre-PSA test stage and as a result, was prone to significant under-staging compared to ProtecT and PIVOT. Secondly, up to 76% of all cases in SPCG-4 are cT2 patients, suggesting a potentially higher risk disease in comparison to other trials. When stratified by risks, OS and CSS are found to be similar amongst those having immediate RP and expectant management in low-risks patients and high-risk patients, but not in intermediate-risk patients. While the results may seem peculiar, it could be likely that patients in the high-risk groups may have had micro-metastasis at the time of enrolment, likely rendering RP to be less effective in controlling PCa disease progression. In summary, the three trials have suggested the oncological safety of active monitoring, watchful waiting or observation in low-risk patients; however, patients must be followed regularly based on annual DRE, PSA and repeated biopsy or MRI to avoid progression [72]. Expectant management of intermediate-risk and high-risk cancer is oncologically unsafe and should be avoided.

In intermediate-risk patients, expectant management should be avoided on the basis of results from the SPCG-4 and the PIVOT trial. Instead, immediate RP, brachytherapy or radiotherapy should be considered in this patient in the first instance. However, when radical treatment is not preferable or suitable, NHT could be considered in such patients. The results of the EORTC 30,891 trial have suggested significantly better overall survival in patients undergoing hormonal therapy immediately compared to deferred hormonal therapy [72]. 

In high-risk patients, the use of NHT should be considered to delay PCa metastasis or progression until surgery is deemed safe to perform. As expected, our study has found oncological outcomes to be similar in those who underwent NHT prior to RP and those who did not. Furthermore, the pathological outcomes are generally excellent after NHT, with significantly lower rates of positive surgical margins, lymph node involvement, pathological upstaging and higher rates of organ confinement and pathological down-staging. Although encouraging, most available evidence reported a 3-month course of NHT. There is insufficient evidence to conclude the safety of 6-month NHT courses.

Conclusions and recommendations regarding the optimal timing of delaying surgery for patients of different PCa risks are difficult to draw. This could be in part due to the significant heterogeneity amongst non-randomised controlled trials. These retrospective studies are hugely limited by different study designs, delay interval cut-offs, lead-time biases, and inadequate follow-up time (>10 years) to allow for accurate evaluation of oncological outcomes [73]. The classification of risks is also varied and unclear in most studies. Nonetheless, a conservative estimate must be taken when decisions are made to delay surgery for PCa patients and hence a delay of less than 5 months, 4 months, and 30 days for low-risk, intermediate-risk, and high-risk patients, respectively, may be acceptable without causing significant harm to the patient without utilising active surveillance strategies. 

Applying the findings of this review to the COVID-19 pandemic, our findings are grossly in concordance with the EAU COVID-19 recommendations [5]. In low-risk patients, the EAU recommended deferring confirmatory re-biopsies and any treatments for up to six months. In intermediate-risk patients, the EAU recommendations suggest treating before the end of 3 months without the use of any NHT. However, studies included in our review have suggested a delay in treatment for intermediate-risk patients for over 4 weeks may result in significantly worsened pathological outcomes [10], hence urologists are advised to carefully balance the risk and benefits of commencing patients on NHT. In high-risk patients, EAU recommends patients to be treated within 3 months (intermediate priority) or 6 weeks (high-priority) and not to use NHT to postpone RP. However, our review suggested a delay of over 30 days may be associated with worsened oncological outcomes [10,21,24,34]. NHT was also shown to successfully delay surgeries for up to 3 months without increased risk of worsened oncological or pathological outcomes; therefore, urologists should also be vigilant in deciding the best treatment or any delay for surgery for high-risk PCa patients. 

While our findings are informative, this study is not without limitations. Most studies included are from the 1980s to the 2000s, before the advancements and popularisation of PSA screening [74], MRI-targeted biopsy [75], and robotic assisted laparoscopic surgery [76]. Modern drugs for NHT now also include newer gonadotropin-releasing hormone antagonists such as degarelix [77]. This may suggest the outcomes of AS, RPs, and NHTs may have significantly advanced since the studies included in this review were published. Nonetheless, this study shall provide valuable information to allow better clinical decision in prioritising patient treatment and backlog clearance in the view of the COVID-19 pandemic. 

## 5. Conclusions

In the case of a delay in surgery, AS and expectant management strategies were found to be generally safe in low-risk PCa patients. In contrast, in intermediate-risk patients, AS and expectant management strategies are less safe. A conservative estimate of maximum acceptable delay treatment for intermediate-risk patients is 4 months, though the evidence is not the strongest. NHT should also be considered carefully in these patients. In high-risk patients, AS and expectant management are inappropriate, thus delay for treatment should be minimised to within 30 days. NHT has been shown to safely delay RP for 3 months without jeopardising oncological and pathological outcomes, and should be considered when postponing treatment. However, there is limited evidence to suggest the safety of NHT therapies for 6 months or longer. Therefore, the use of NHT to delay surgery for over 3 months must be carefully considered. 

## Figures and Tables

**Table 1:** Summary of meta-analyses comparing 3 months of NHT and delayed RP versus immediate RP.

Outcome	No. of Studies	Risk Ratio	95% CI	*p*-Value
Overall Deaths	4	1.30	0.81–2.09	0.28
Cancer-specific Deaths	4	1.25	0.46–3.40	0.66
Positive Surgical Margin	10	0.45	0.37–0.54	<0.01
Organ Confinement	6	1.39	1.10–1.76	<0.01
Lymph node involvement	6	0.68	0.48–0.97	0.03
Seminal Vesicle involvement	3	1.02	0.44–2.32	0.97
Pathological Upstaging	2	0.51	0.34–0.76	<0.01
Pathological Down-staging	3	2.38	1.49–3.78	<0.01
PSA Failure	5	0.96 (HR)	0.78–1.19	0.72

CI: Confidence Interval; PSA: Prostate specific antigen; HR: Hazard Ratio.

## Data Availability

This study is a review and all data used are available in literature.

## Supplementary Materials

The following are available online at https://www.mdpi.com/article/10.3390/cancers13133274/s1, Table S1: A summary of baseline characteristics of included RCTs comparing expectant management and immediate RP; Figure S1: Risk of Bias of included RCTs comparing expectant management and immediate RP; Figure S2: Sensitivity analysis of overall Survival in expectant management patients and immediate RP patients in RCTs; Figure S3: Sensitivity analysis of cancer-specific survival in expectant management patients and immediate RP patients in RCTs; Figure S4: Progression Free Survival in expectant management patients and immediate RP patients in RCTs; Figure S5: Overall Survival in risk stratified expectant management patients and immediate RP patients in RCTs; Figure S6: Cancer-specific Survival in risk-stratified expectant management patients and immediate RP patients in RCTs; Figure S7: Progression-free survival in risk-stratified expectant management patients and immediate RP patients in RCTs; Table S2: A summary of baseline characteristics of included observational studies comparing delayed RP and immediate RP; Table S3: Risk of bias assessment of observational studies comparing delayed RP and immediate RP; Table S4: A summary of baseline characteristics and outcomes of included RCTs comparing NHT and immediate RP; Figure S8: Overall deaths in patients undergoing 3 months of NHT followed by RP and immediate RP; Figure S9: Cancer-specific deaths in patients undergoing 3 months of NHT followed by RP and immediate RP; Figure S10: seminal vesicle involvement in patients undergoing 3 months of NHT followed by RP and immediate RP; Figure S11: pathological upstaging in patients undergoing 3 months of NHT followed by RP and immediate RP; Figure S12: pathological downstaging involvement in patients undergoing 3 months of NHT followed by RP and immediate RP; Figure S13: PSA failure involvement in patients undergoing 3 months of NHT followed by RP and immediate RP; Table S4: A summary of baseline characteristics and outcomes of non-randomised studies comparing NHT and immediate RP; Table S5: Risk of bias of non-randomised studies comparing NHT and immediate RP.
