# Supplementary material for: Effects of Delayed Radical Prostatectomy and Active Surveillance on Localised Prostate Cancer—A Systematic Review and Meta-Analysis
Source: Cancers (Basel). 2021 Jun 30;13(13):3274. doi: 10.3390/cancers13133274 (PMC8268689; doi:10.3390/cancers13133274)
Supplement: Supplementary file 1 [file cancers-13-03274-s001.zip › cancers-1237809-supplementary.pdf]

Supplementary Material to "Delayed radical prostatectomy for prostate cancer: A systematic review".

Authors: Vinson Wai-Shun Chan*, Wei Shen Tan*, Aqua Asif, Alexander Ng, Olayinka Gbolahan, Eoin Dinneen, Wilson To, Hassan Kadhim, Melissa Premchand, Oliver Burton, Jasmine Sze-Ern Koe, Nicole Wang, Jeffrey Leow, Gianluca Giannarini, Nikhil Vasdev, Shahrokh F. Shariat, Dmitry Enikeev, Chi Fai Ng, Jeremy Yuen-Chun Teoh

Contents

Table S1 – A summary of baseline characteristics of included RCTs comparing expectant management and immediate RP 3

Figure S1 – Risk of Bias of included RCTs comparing expectant management and immediate RP 4

Figure S2 – Sensitivity analysis of overall Survival in expectant management patients and immediate RP patients in RCTs 5

Figure S3 – Sensitivity analysis of cancer-specific survival in expectant management patients and immediate RP patients in RCTs 6

Figure S4 –Progression Free Survival in expectant management patients and immediate RP patients in RCTs 7

Figure S5 –Overall Survival in risk stratified expectant management patients and immediate RP patients in RCTs 8

Figure S6 – Cancer-specific Survival in risk-stratified expectant management patients and immediate RP patients in RCTs 9

Figure S7 – Progression-free survival in risk-stratified expectant management patients and immediate RP patients in RCTs 10

Table S2 – A summary of baseline characteristics of included observational studies comparing delayed RP and immediate RP 11

Table S3 – Risk of bias assessment of observational studies comparing delayed RP and immediate RP 17

Table S4 – A summary of baseline characteristics and outcomes of included RCTs comparing NHT and immediate RP 21

Figure S8 – Overall deaths in patients undergoing 3-months NHT followed by RP and immediate RP 24

Figure S9 – Cancer-specific deaths in patients undergoing 3-months NHT followed by RP and immediate RP 25

Figure S10 – seminal vesicle involvement in patients undergoing 3-months NHT followed by RP and immediate RP 26

Figure S11 – pathological upstaging in patients undergoing 3-months NHT followed by RP and immediate RP 27

Figure S12 – pathological downstaging involvement in patients undergoing 3-months NHT followed by RP and immediate RP 28

Figure S13 – PSA failure involvement in patients undergoing 3-months NHT followed by RP and immediate RP 29

Table S5 – A summary of baseline characteristics and outcomes of non-randomised studies comparing NHT and immediate RP 30

Table S6 – Risk of bias of non-randomised studies comparing NHT and immediate RP 32

Table S1 – A summary of baseline characteristics of included RCTs comparing expectant management and immediate RP

Study	Year of study	Number of patients (AS/RP)	Recruitment Period	Country of study	Intervention	AS/WW Protocol	Comparator	Follow-up protocol	Median / mean follow-up period	Median / mean age	Median (IQR)/ mean (range) PSA	cT stage	GG score	Disease risk
**SPCG-4**	2014	348/347	October 1989 and December 1999	Sweden, Finland, and Iceland	Watchful waiting	No immediate treatment (apart from TUR-P)	Immediate RP	Every 6 months for 2 years and annually thereafter<br>PSA Bone Scan Chest X-ray Prostate Ultrasound annually	Median (range): 13.4 (3 weeks to 23.2) years)	Mean (SD)<br>WW: 64.5 (5.0)<br>RP: 64.6 (5.1)	Mean<br>WW: 12.3<br>RP: 13.5	WW:<br>T1b: 50<br>T1c: 38<br>T2: 259<br>Unknown: 1<br><br>RP:<br>T1b: 33<br>T1c: 43<br>T2: 270	WW:<br>2-4: 46<br>5-6: 166<br>7: 82<br>8-10: 21<br>Unknown: 33<br><br>RP:<br>2-4: 45<br>5-6: 165<br>7: 77<br>8-10: 14<br>Unknown: 46	WW:<br>Low: 131<br>Intermediate: 133<br>High: 84<br><br>RP:<br>Low: 118<br>Intermediate: 148<br>High: 81<br><br>Definitions:<br>Low risk: PSA < 10ng/ml and Gleason score <7 or WHO grade 1<br>Intermediate risk: Patients not fulfilling criteria for low or high risk<br>High risk: PSA > 20 ng/ml or Gleason score > 7
**PIVOT**	2017	367/363	November 1994 through January 2002	United States of America	Observation	Therapy only reserved for symptomatic or metastatic disease<br>Asymptomatic disease progression = not treated	Immediate RP	Every 3 months for first year and every 6 months thereafter. DRE, Bone scans, PSA, PAP	Median (IQR): 12.7 (7.3 to 15.5)	Mean: 67	Median: 7.8 ng per milliliter	NR	Obs:<br><7: 261<br>≥7 86<br><br>RP:<br><7: 254<br>≥7 98	D'Amico<br>Obs:<br>Low: 148<br>Intermediate: 120<br>High: 80<br><br>RP:<br>Low: 148<br>Intermediate: 129<br>High: 77
**ProtecT**	2016	545/553	1999 and 2009	United Kingdom	Active Monitoring	Suspected progression triggers review of treatment options	Immediate RP	Every 3 months for first year and annually thereafter. PSA DRE	Median: 10	Mean (SD)<br>AM: 62 (5)<br>RP: 62 (5)	Median (IQR)<br>ng/mnl<br>AM: 4.7 (3.7, 6.7)<br>RP: 4.9 (3.7, 6.7)	AM:<br>T1c: 410<br>T2: 135<br><br>RP:<br>T1c: 410<br>T2: 143	AM:<br>6: 421<br>7:111<br>8-10: 13<br><br>RP:<br>6: 422<br>7: 120<br>8-10: 10	NR
**VACURG**	1990		May 1967 and March 1975	United States of America	Placebo	NR	Immediate RP	At six months intervals	Median: 15 years	Stage 1<br>Placebo<br><60: 5<br>60-69: 9<br>>70: 16<br>RP + Placebo<br><60: 11<br>60-69: 9<br>>70: 11<br><br>Stage 2<br>Placebo<br><60: 7<br>60-69: 8<br>>70: 5<br>RP + Placebo<br><60: 11<br>60-69: 13<br>>70: 6	N/A	UICC<br><br>Placebo<br>T0: 39<br>T1/2: 37<br><br>RP<br>T0: 37<br>T1/2: 37	Placebo:<br>5-6: 33<br>7-10: 3<br>Unknown: 3<br><br>RP:<br>5-6: 42<br>7-10: 7<br>Unknown: 3	NR

Figure S1 – Risk of Bias of included RCTs comparing expectant management and immediate RP

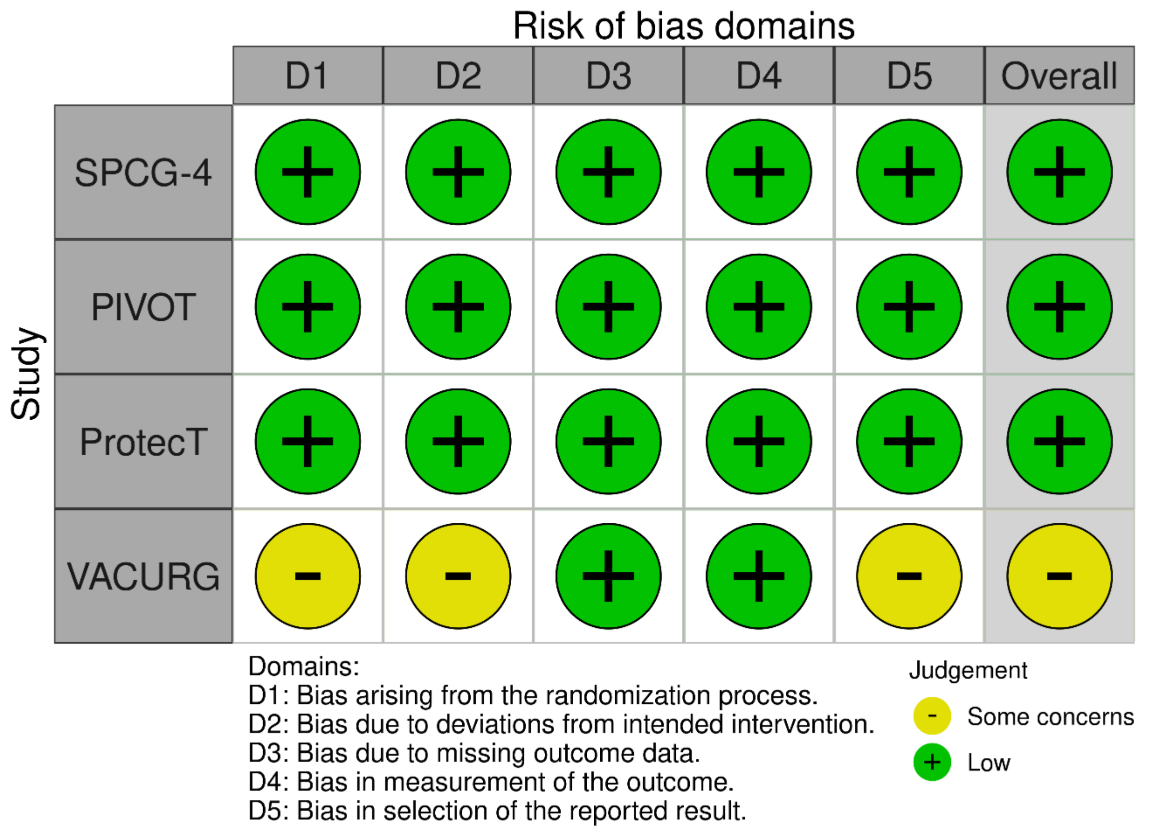

(a) Individual Risk of bias of included studies

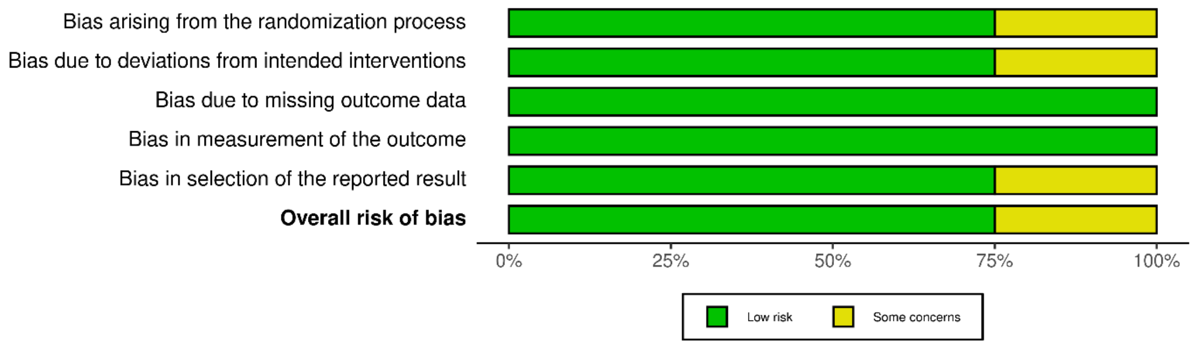

(b) Summary of risk of bias in included studies

Figure S2 – Sensitivity analysis of overall Survival in expectant management patients and immediate RP patients in RCTs

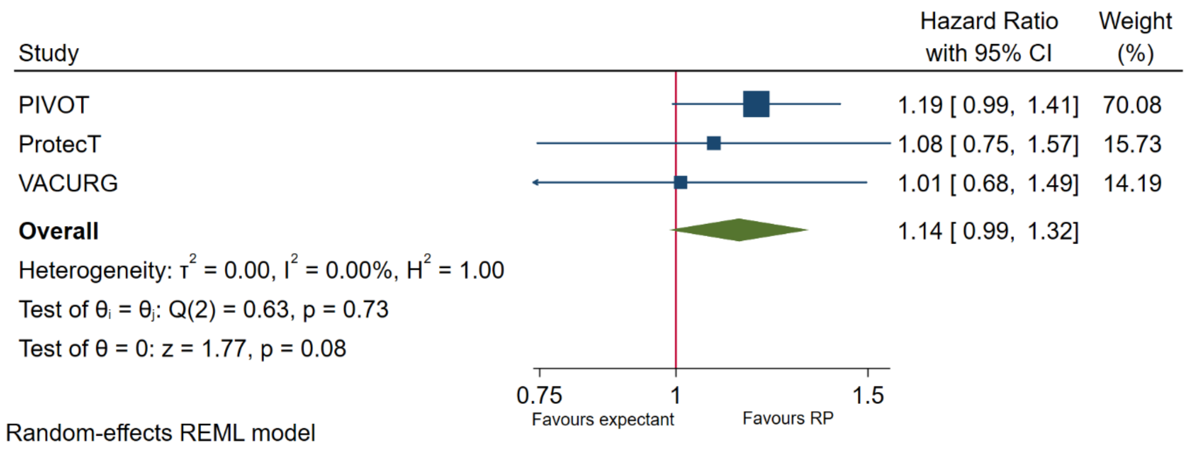

Figure S3 – Sensitivity analysis of cancer-specific survival in expectant management patients and immediate RP patients in RCTs

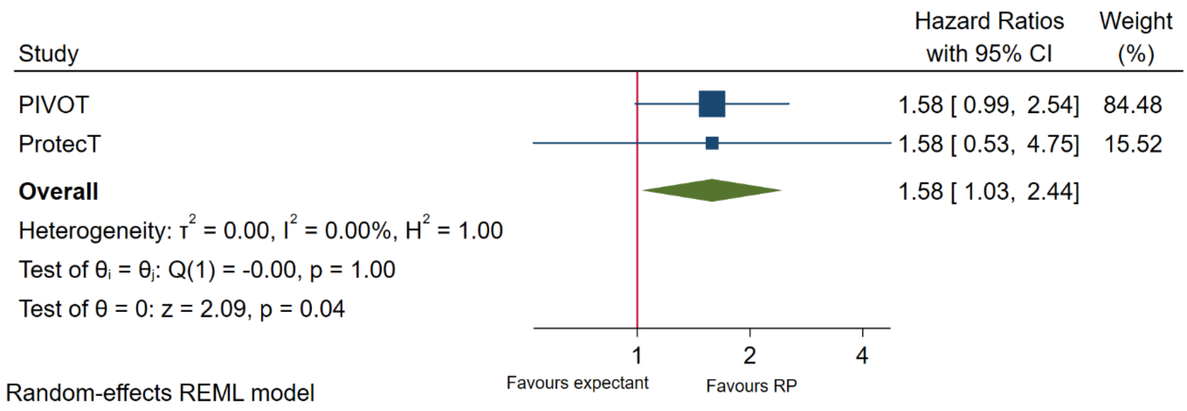

Figure S4 –Progression Free Survival in expectant management patients and immediate RP patients in RCTs

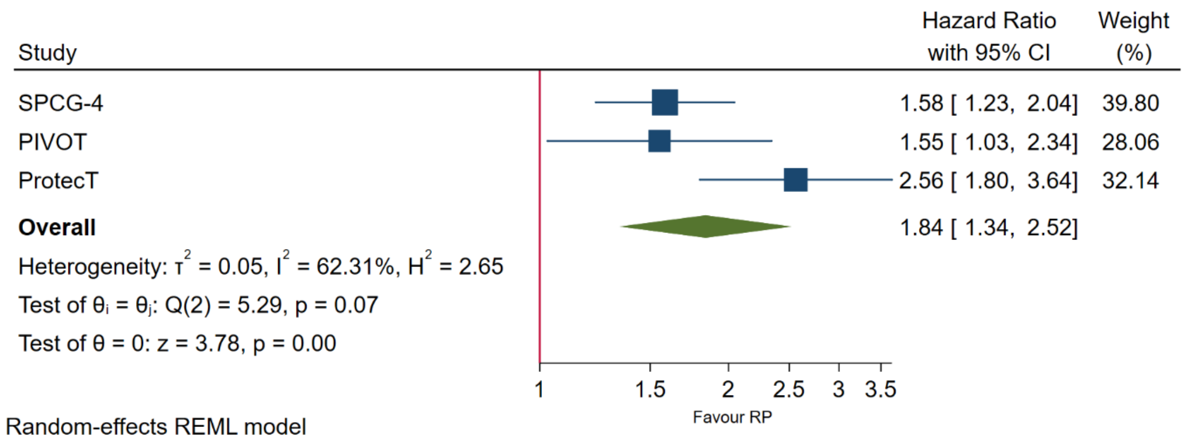

Figure S5 –Overall Survival in risk stratified expectant management patients and immediate RP patients in RCTs

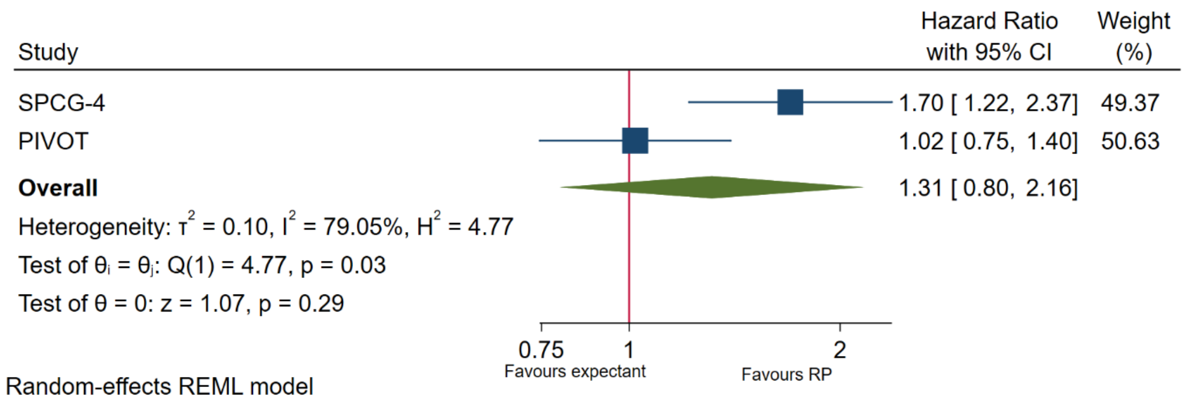

(a) Low-risk

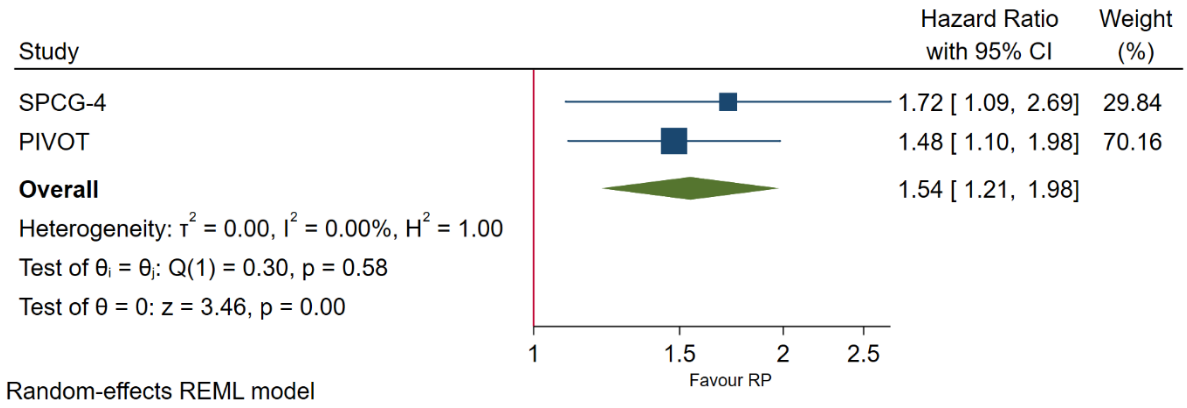

(b) Intermediate-risk

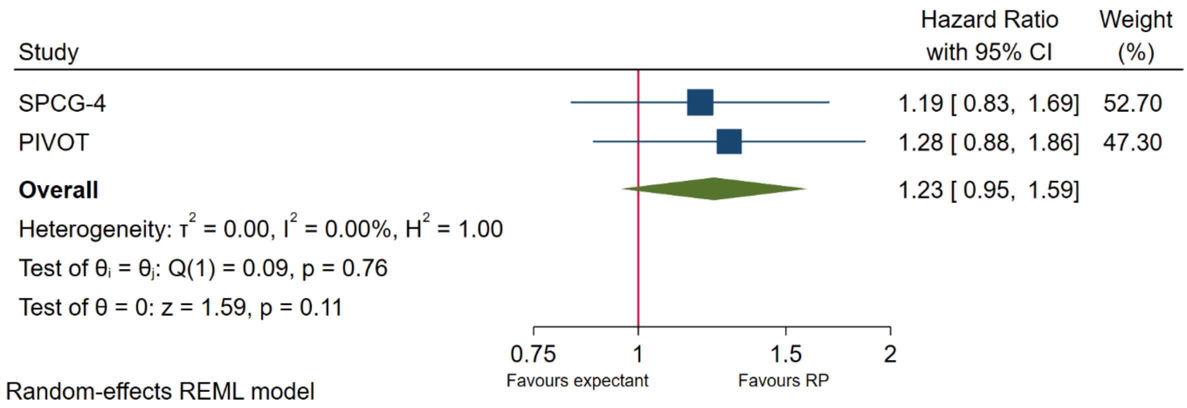

(c) High-risk

Figure S6 – Cancer-specific Survival in risk-stratified expectant management patients and immediate RP patients in RCTs

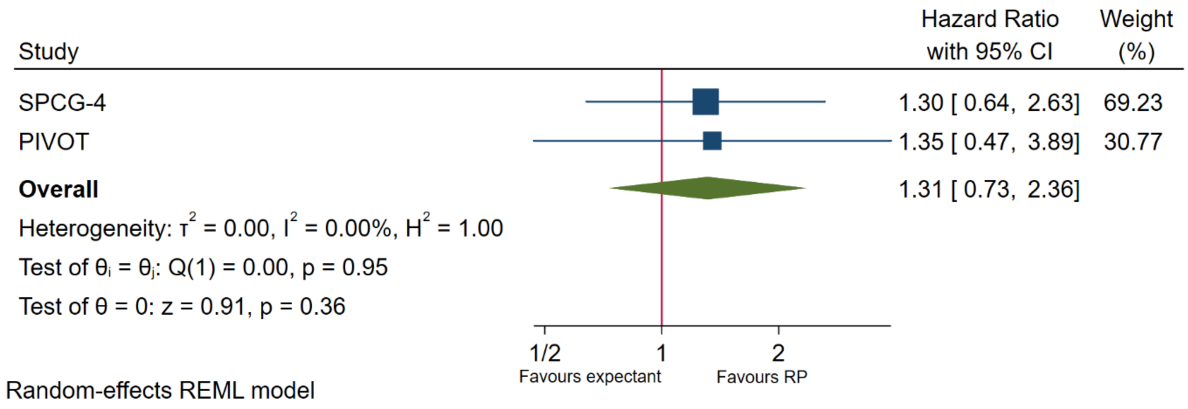

(a) Low-risk

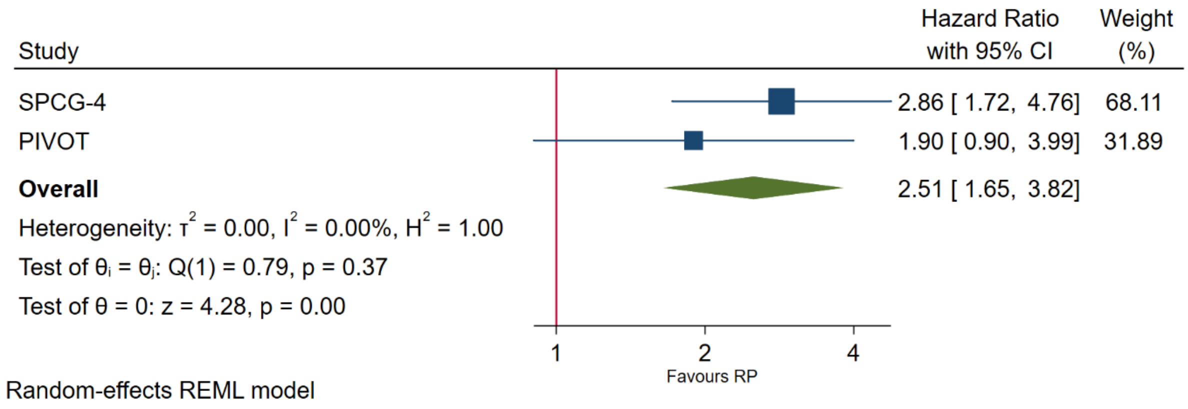

(b) Intermediate-risk

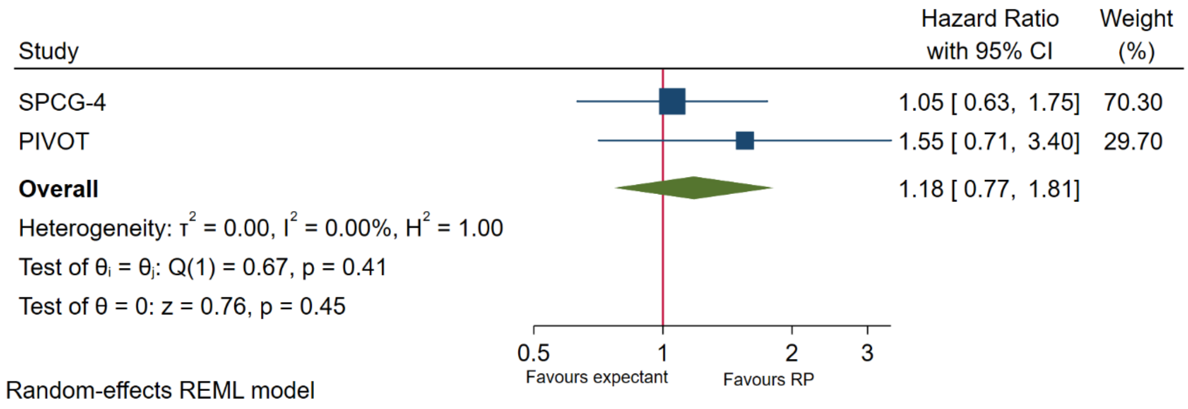

(c) High-risk

Figure S7 – Progression-free survival in risk-stratified expectant management patients and immediate RP patients in RCTs

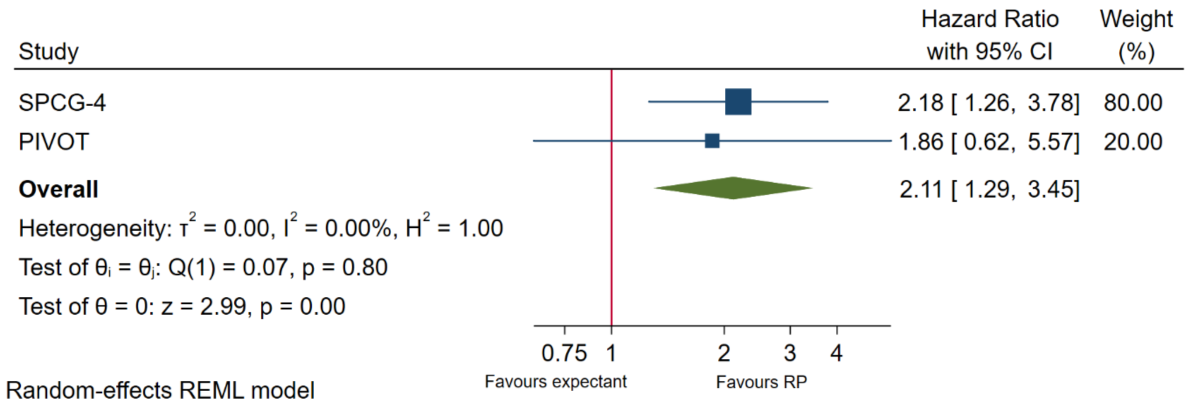

(a) Low-risk

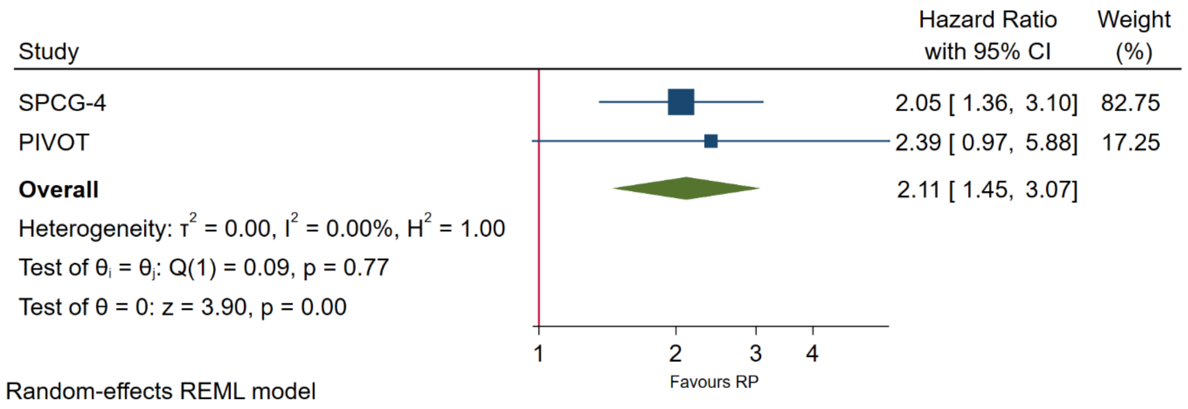

(b) Intermediate-risk

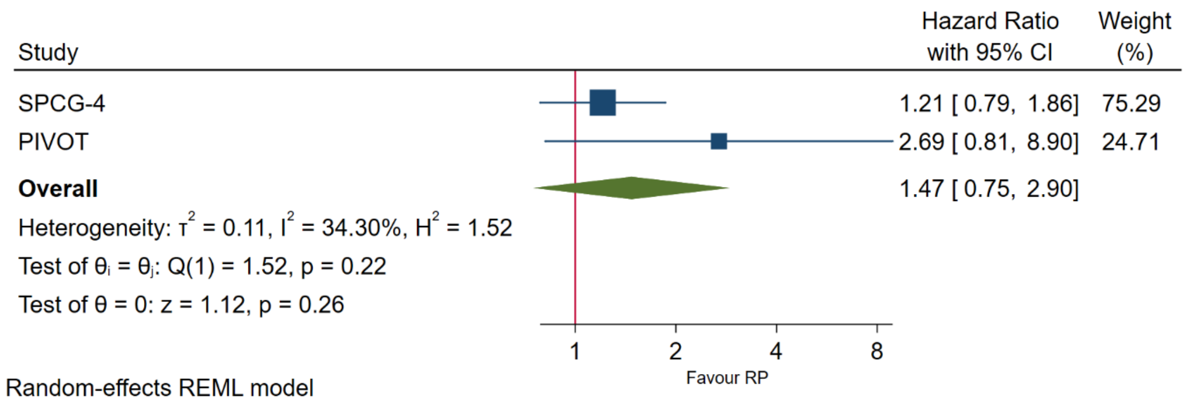

(c) High-risk

Table S2 – A summary of baseline characteristics of included observational studies comparing delayed RP and immediate RP

Study	Inclusion criteria	Exclusion criteria	Study design number of patients	Definition of delayed surgery / duration of delay	Year of treatment	Median (IQR) / mean (range) follow-up	Median (IQR)/ mean (range) age	Median (IQR)/ mean (range) PSA	cT stage	GG score	Disease risk	RFS / BRFS	PFS / BPFS	CSS / OS	Outcome
**Awasthi 2019**	Histologically confirmed localized PCa<br>No neoadjuvant treatment<br>Not under active surveillance		Retrospective two centre cohort:<br>Total: 1,807 men<br>0-3: 1,111 men<br><br>562 men<br><br>134 men	0-3 months delay<br><br>3-6 months delay<br><br>>6 months delay	1987-2015	Median: 46 months<br>IQR: 18-86	Median age: 60<br>IQR: 55-64	0-6: 57.5%<br>6-10: 25.9%<br>10-20: 13.0%<br>>20: 3.5%<br>0-6: 56.0%<br>6-10: 29.5%<br>10-20: 11.7%<br>>20: 2.7%<br>0-6: 52.2%<br>6-10: 29.8%<br>10-20: 12.7%<br>>20: 3.2%	T1: 62.9%<br>T2: 21.4%<br>T3: 1.4%<br>Unknown: 14.2%<br>T1: T1: 64.4%<br>T2: 21.3%<br>T3: 0.7%<br>Unknown: 13.5%<br>T1: 60.4%<br>T2: 25.4%<br>T3: 0%<br>Unknown: 14.2%	GG1: 62.9%<br>GG2 & 3: 48.1%<br>GG4: 4.1%<br>GG5: 2.9%<br>GG1: 49.5%<br>GG2-3: 45.0%<br>GG4: 3.2%<br>GG5: 2.3%<br>GG1: 46.3%<br>GG2-3: 45.0%<br>GG4: 6.0%<br>GG5: 2.2%	NCCN<br><br>Low: 47.6%<br>Int: 34.6%<br>High: 9.7%<br>Low: 55.5%<br>Int: 30.8%<br>High: 6.0%<br><br>Low: 54.5%<br>Int: 31.3%<br>High: 8.2%	5-year:<br>0-3-mo: 78% (95%CI: 75-81%)<br><br>3-6-mo: 82% (78-85%)<br><br>>6-mo: 69% (59%-77%)	NR	NR	Delay of >6 month was associated with BCR in **intermediate** risk patients only<br><br>No difference in RP Gleason score/ upgrade, extracapsular extension, PSM in patients with delayed RP overall
**Patel 2019**	Undergoing RP		Retrospective single centre:<br><br>2,310 men	<2 months<br><br>2-3 months<br><br>3-4 months<br><br>4-5 months<br><br>5-6 months	2005 – 2014	Time of diagnosis to RP	Mean: 60.2<br>Range: 38.8-84.2	Median IQR):<br>6.0 (4.7-8.1)	NR	GG1: 39%<br>GG2: 45%<br>GG3: 10%<br>GG4: 3%<br>GG5: 2.4%	NR	NR	NR	NR	No difference was observed for patients in any risk category regarding the adverse pathologic outcomes, including GG upgrade from biopsy to RP, EPE, SVI, PSM, and positive LN involvement, with delays of ≤ 6 months
**Westerman 2018**	cT1 - 3N0M0 PCa<br>Prostate biopsy within one year<br>No neoadjuvant therapy		Retrospective single centre<br><br>7,350 men	Overall<br><br><3 weeks<br><br>4-6 weeks<br><br>7-12 weeks<br><br>12-26 weeks<br><br>>26 weeks	1994 – 2012	Median 7.1 years<br>IQRL 4.2-11.7	Mean: 61.5<br>SD 7.1<br><br>Mean: 63.3<br>SD:7.3<br><br>Mean: 62.7<br>SD: 7.1<br><br>Mean: 60.8<br>SD:7.1<br><br>Mean: 61.0<br>SD:6.8<br><br>Mean: 62.3<br>SD:6.3	Mean: 7.1<br>SD:7.3<br><br>Mean: 8.6<br>SD:11.7<br><br>Mean: 8.3<br>SD: 9.0<br><br>Mean: 6.7<br>SD: 5.9<br><br>Mean: 6.1<br>SD: 4.6<br><br>Mean: 6.5<br>SD: 5.0	cT1: 60.2%<br>cT2: 38.2%<br>cT3: 1.6%<br>cT1: 44.5%<br>cT2: 53.3%<br>cT3: 2.2%<br>cT1: 48.4%<br>cT2: 47.9%<br>cT3: 3.8%<br>cT1: 63.6%<br>cT2: 35.2%<br>cT3: 1.2%<br>cT1: 69.5%<br>cT2: 30.0%<br>cT3: 0.5%<br><br>cT1: 74.3%<br>cT2: 24.8%<br>cT3: 1%	GG1: 69%<br>GG2-3: 25.8%<br>GG4-5: 5,2%<br>GG1: 68.5%<br>GG2-3: 22.2%<br>GG4-5: 9.3%<br>GG1: 61.9%<br>GG2-3: 29.5%<br>GG4-5: 8.6%<br>GG1: 67.6%<br>GG2-3: 27.8%<br>GG4-5: 4.6%<br>GG1: 75.3%<br>GG2-3: 22.6%<br>GG4-5: 2.1%<br><br>GG1: 85.2%<br>GG2-3: 13.3%<br>GG4-5: 1.4%	NCCN<br>Low: 53.6%<br>Int: 37.7%<br>High: 8.7%<br>Low: 43%<br>Int: 41.6%<br>High: 15.3%<br>Low: 41.9%<br>Int: 43.4%<br>High: 14.7%<br>Low: 54.5%<br>Int: 38.2%<br>High: 7.4%<br>Low: 64.4%<br>Int: 31.8%<br>High: 3.8%<br><br>Low: 69.5%<br>Int: 26.7%<br>High: 3.8%	(HR (95% CI))<br><br>Low Risk Group:<br>Delay 7-12 weeks (HR 1.64, 1.26-2.12)<br>Delay 13-26 weeks (HR 1.69, 1.25-2.28)<br>Delay 26-52 weeks: NS<br><br>Int Risk Group:<br>NS<br><br>High Risk Group:<br>Delay 26-52 weeks (HR 3.03, 1.05-8.78)	NR	NR	**Low risk**:<br>Delay in >7 weeks associated with BCR but not delay in 26-52 weeks<br>Delay >7 weeks associated with higher upgrading. No difference in ≥pT3-4, PSM<br><br>**Int risk**:<br>BCR NS delay RP<br>Delay >4 weeks associated with upgrading. No No difference in ≥pT3-4, PSM<br><br>**High risk**:<br>Delay in 26-52 weeks associated with BCR<br>No difference delay in RP and upgrading, ≥pT3-4, PSM
**Anil 2018**	NR		Retrospective<br><br>248	Overall<br><br>0-60 days<br><br>61-120 days<br><br>>121 days	2008-2017	Mean: 16.1 months<br>Range 2-72 months	Mean 64.2<br>SD no given<br><br>Mean 64.2<br>SD: 6.5<br><br>Mean 64.1<br>SD: 6.7<br><br>Mean: 65.3<br>SD: 5.7	Mean 9.96<br>SD not given<br><br>Median: 7.3<br>Range:3.5-52.6<br><br>Median: 7.7<br>Range: 3.2-55<br><br>Median: 7.2<br>Range: 2.3-38	cT1: 39%<br>cT2: 61%<br>-seems no cT3<br><br>cT1: 41.1%<br>cT2: 58.9%<br><br>cT1: 31.9%<br>cT2: 68.1%<br><br>cT1: 64.3%<br>cT2: 35.7%	GG1:70.9%<br>GG2-3: 22.6%<br>GG4-5: 6.4%<br><br>GG1:73.8 %<br>GG2-3: 21.5%<br>GG4-5: 4.7%<br><br>GG1:66.4%<br>GG2-3: 24.8%<br>GG4-5: 8.8%<br><br>GG1:78.6%<br>GG2-3: 17.9%<br>GG4-5: 3.6%	Amico/Overall<br>Low: 122 (49%)<br>Int: 63 (25.4%)<br>High: 63 (25.4%)<br>Low: 53%<br>Int: 21.5%<br>High: 25%<br><br>Low: 43%<br>Int: 29.2%<br>High: 27.4%<br><br>Low: 57.1%<br>Int: 25%<br>High: 17.9%	Overall<br><br>79.8%<br><br>72.4%<br><br>69.4%<br><br>100%	NR	NR	No differences in pathological outcome and pathological outcome after 60 days+ delay in low risk and high risk patients<br><br>In the Int group, the rate of extraprostatic extension was significantly higher as surgical delay increased (p=0.044)
**Gupta 2019**	Clinically localised PCa for RP GG1/2<br>RP within 6 months of diagnosis		2303	Overall<br><br>0-3 months (n=1658)<br><br>3-6 months (n=645)	2005-2018	Median: 3yrs<br>IQR: 2-5yrs	GG3<br><br>Median (IQR): 62 (57-66)<br><br>GG4<br><br>Median (IQR): 63 (57-66)<br><br>GG5<br><br>Median (IQR): 63 (57-67)	GG3<br><br>Median (IQR): 5.8 (4.5 – 8.3)<br><br>GG4<br><br>Median (IQR): 6.2 (4.6-9.6)<br><br>GG5<br><br>Median (IQR): 6.4 (4.7-10.2)	GG3:<br><br>cT1c: 852 (69%)<br>cT2a: 226 (!8%)<br>cT2b: 140 (11%)<br>cT2c-cT3: 26 (2%)<br><br>GG4:<br><br>cT1c: 362 (60%)<br>cT2a: 134 (22%)<br>cT2b: 85 (14%)<br>cT2c- cT3: 27 (4%)<br><br>GG5:<br><br>cT1c: 225 (50%)<br>cT2a: 100 (27%)<br>cT2b: 79 (18%)<br>cT2c- cT3:47(10%)	Overall:<br><br>GG3: 1244 (54%)<br><br>GG4: 608 (26.4%)<br><br>GG5: 451 (19.6%)	NR	Overall<br>*adjuvant Tx<br><br>70%<br><br>Breakdown given only in terms of their Gleason Group<br>GG3:76%<br>GG4: 64%<br>GG5: 56%<br><br>GG3: 80%<br>GG4: 69%<br>GG5: 60%	Overall<br>*MFS<br><br>2yr: 98%<br>5yr: 93%<br>10yr: 86%<br><br>2yr: 98%<br>5yr:92%<br>10yr: 84%<br><br>2yr: 97%<br>5yr: 95%<br>10yr: 91%	NR	No significant difference in the proportion who received adj therapy between men who received RP <3 vs. 3-6 months in GG 3, 4, and 5<br><br>There were no significant differences in 2 and 5 yr BCRFS rates between men who underwent RP<3months vs. 3-6 months in GG 3, 4, and 5<br><br>There were no significant differences in pathological outcome and adverse events postoperatively between men who underwent RP<3 vs. 3-6 months in GG 3,4, and 5<br><br>There was no significant difference in MFS rates

														between men who received RP <3month vs 3-6 months in all GGs
**Aas 2018**	Non metastatic PCa<br>RP within 180 days of first positive biopsy.<br>PSA< 100 ng/ml<br>GS >= 5	Retrospective national cancer registry cohort study<br><br>5163	Overall	2001-2010	Median 7.9yrs<br>Range 0-15yrs	Median: 62<br>Range: 39-77	<10: 69.4%<br>10-20: 25.7%<br>>20: 5%	cT1: 47%<br>cT2: 44.2%<br>cT3-4: 8.7%	GG1:47.1%<br>GG2: 31.4%<br>GG3: 12.2%<br>GG4: 7.2%<br>GG5: 2.1%	EAU Guidelines<br>Low: 28.2%<br>Int: 42.9%<br>H local: 20%<br>H local adv.: 8.8%	"RP failure"<br><br>24.7%<br><br>*see Figure 2 for KM's.<br><br>In all risk groups, increasing RP-interval was significantly associated with decreased probability of RP-failure	NR	PCSM<br><br>1.9%	In **int, high and high locally advanced** there were no significant differences in PCSM according to RP interval
			0-60 days (17%)			Median: 62<br>Range:39-75	<10: 69.6%<br>10-20: 25.6%<br>>20: 4.8%	cT1: 44.8%<br>cT2: 47%<br>cT3-4: 8.2%	GG1: 45.1%<br>GG2: 28.5%<br>GG3: 13.6%<br>GG4:10.8%<br>GG5: 2.1%	Low: 28.3%<br>Int: 40.6%<br>H local: 22.8%<br>H local adv.: 8.2%			2.1%	No associations between length of the RP interval and the rate of upgrading, upstaging, or positive margins.<br><br>In **all risk groups**, increasing RP-interval was significantly associated with decreased probability of RP-failure. This find- ing may indicate that patients with shorter RP-intervals had more aggressive disease than reflected by our co-variates (e.g., the number of biopsy cores involved with cancer, the percentage of cancer within each biopsy core, PSA doubling time)
			61-90 days (31%)			Median: 62<br>Range: 39-76	<10: 69.6%<br>10-20: 25.8%<br>>20: 4.7%	cT1: 45.3%<br>cT2: 45%<br>cT3-4: 9.7%	GG1: 46.8%<br>GG2: 31.7%<br>GG3: 11.9%<br>GG4: 7.2%<br>GG5: 2.5%	Low: 27.8%<br>Int: 42.7%<br>H local: 19.8%<br>H local adv.: 9.7%			2.7%	
			91-120 days (26%)			Median: 62<br>Range: 42-77	<10: 71.7%<br>10-20: 23.5%<br>>20: 4.8%	cT1: 46.7%<br>cT2:44.5%<br>cT3-4: 8.7%	GG1: 45.0%<br>GG2: 34.1%<br>GG3: 11.9%<br>GG4: 6.8%<br>GG5: 2.2%	Low: 28%<br>Int: 44.1%<br>H local: 19.1%<br>H local adv.: 8.8%			1.5%	
			121-180 days (27%)			Median: 62<br>Range: 42-76	<10: 66.8%<br>10-20: 27.6%<br>>20: 5.6%	cT1: 50.5%<br>cT2: 41.2%<br>cT3-4: 8.4%	GG1: 50.8%<br>GG2: 30.5%<br>GG3: 11.8%<br>GG4: 5.3%<br>GG5: 1.6%	Low: 28.8%<br>Int: 43.4%<br>H local: 19.4%<br>H local adv.: 8.4%			1.3%	
**O'Callaghan 2017**	NR	Retrospective Regional registry cohort study<br><br>3140	Quartiles of delay<br>Overall	1998-2013	NR	Mean: 66.7<br>SD:9.2	Median: 9.1<br>IQR: 6.3-15.7	NR	GG1: 43.1%<br>GG2: 24.8%<br>GG3: 13.1%<br>GG4-5: 15.7%	NR	NR	NR	OS:83.6%<br>CSS: 93%	Shorter delays associated with higher prostate cancer mortality (HR 2.37), this remained after adjustment for age, GG at biopsy, PSA.<br><br>Increased delays did not appear to influence overall survival or prostate cancer specific mortality after adjustment for patient characteristics. On the contrary shorter delays were associated with poorer outcomes
			Q1: 0-35 days (793)			Mean: 70.2<br>SD:10.4	Median: 14.9<br>IQR:7.7-42.5		GG1: 31.7%<br>GG2: 19.5%<br>GG3: 13.2%<br>GG4-5: 28.9%				OS: 62.2%<br>CSS: 82.3%	
			Q2: 36-86 days (793)			Mean: 64.9<br>SD: 8.0	Median: 8.3<br>IQR:5.9-12		GG1: 42.7%<br>GG2: 28.8%<br>GG3: 14.9%<br>GG4-5: 11%				OS: 90%<br>CSS: 97.1%	
			Q3: 87-138 days (773)			Mean: 65<br>SD: 7.9	Median: 8<br>IQR:6.1-11.7		GG1: 46.7%<br>GG2: 29.2%<br>GG3: 12.2%<br>GG4-5: 10.1%				OS: 92.5%<br>CSS: 96.7%	
			Q4: 139-264 days (781)			Mean: 66.7<br>SD: 8.3	Median: 8.6<br>IQR:6.3-13.8		GG1:51.5%<br>GG2: 21.8%<br>GG3: 12%<br>GG4-5: 12.8%				OS: 91.2%<br>CSS: 96%	
**Zanaty 2017**	Patients undergoing RP for PCa<br>No evidence of residual disease post-operatively<br>PSA>0.1 ng/ml post operatively<br>Not received NHT	Retrospective two centre cohort<br><br>619	Mean 'Surgical Wait Time' (SWT) 153 days<br><br>Mean SWT 169, 150, 125 days for low, int and high-risk disease, respectively	2006-2015	Mean 28 months<br>Median 22 months	3 groups by D'Amico:<br><br>Low Risk:<br>Mean: 58.32yrs	3 groups by D'Amico:<br><br>Low risk<br>Mean: 4.95	3 groups by D'Amico:<br><br>Low Risk<br>cT1:<br>cT2: 100%<br>cT3:	3 groups by D'Amico:<br><br>Low Risk<br>GG1: 100%<br>GG2<br>GG3<br>GG4-5	n/a	Hazard Ratio for delay by multi variate cox regression analysis:<br><br>Overall:<br><br>HR: 1 (CI: 0.997-1.004)<br><br>Low:<br>HR: 0.957 (0.91-1.006)	NR	NR	On analysis of the entire cohort with multi-variable analysis delay did not affect BCR<br><br>In high-risk group delay was significantly associated with BCR in both univariate and multivariate analysis<br><br>KM curve showed significance for BCR in high-risk patients with a cut off of 90 days
						Int Risk<br>Mean: 60.65yrs	Int Risk<br>Mean: 6.41	Int Risk<br><br>cT1: 71.5%<br>cT2: 28.5%<br>cT3:	Int Risk<br><br>GG1: 5%<br>GG2: 75%<br>GG3: 20%<br>GG4-5:		Int Risk<br><br>HR: 1 (0.996-1.005)			
						High Risk<br>Mean: 63.8yrs	High Risk<br>Mean: 10.92	High Risk<br><br>cT1: 43.6%<br>cT2: 46.5%<br>cT3: 9.85%	High Risk<br><br>GG1: 1.4%<br>GG2: 8.45%<br>GG3:5.63%<br>GG4-5: 84.5%		High Risk<br><br>HR: 1.016 (1.006-1.026)			
**Filippou 2015**	No previous treatment for PCa<br>Active Surveillance<br>No NHT received	Retrospective single centre cohort<br><br>678	RP < 6-mo from first biopsy (n=521)	1990-2014	Median: 33<br>IQR: 10-67	Mean: 58.8<br>SD: 6.8	Median: 5.4<br>IQR: 4.3-6.6	cT2: 44%<br>(nil else given)	NR	CAPRA<br><br>Low: 100%	BRFS: 96%<br><br>Upgrade to G4+3 or above: 5%<br>EPE: 11%<br>PSM: 11%	NR	NR	In **low-risk** patients, delay > 6 months associated with worsened GS upgrade and cancer volume increase, extraprostatic extension and positive surgical margins. BCR or additional treatment significantly worse in >6 months delay, however, no longer significant after matching
			Delayed RP >6months from first biopsy (n=157)		Median: 40<br>IQR: 25-62	Mean: 60.6<br>SD: 6.9	Median: 4.9<br>IQR: 4-6.1	cT2: 34%<br>(nil else given)		Low: 100%	BRFS: 93%<br>Upgrade to G4+3 or above: 12%<br>EPE: 25%<br>PSM: 21%			
**Fossati 2017**	RP for PCa<br>No prior radiation therapy or hormonal treatment	Retrospective single centre cohort	Median delay: 2.8 months (1.6-4.7)	2006-2011	Median 56 months (26-92)	Median: 66 (60-70)	Median: 6.5 (4.8-7.1)	cT1:59%<br>cT2: 32%<br>cT3: 9%	GG1: 63%<br>GG2-3: 32%<br>GG3-4: 5%	EAU Guidelines<br>Low: 35%<br>Int: 50%				Delay was associate with BCR in **high-risk** (HR = 1.05; 95% CI: 1.02,

	Not managed using Active Surveillance	2,653 RP								High: 15%				1.08; P = 0.0001) and CR (HR = 1.06; 95% CI: 1.03, 1.09; P = 0.0001) but now **low- and intermediate-risk**
**Loeb 2016**	Age < 70<br>Clinical stage T1/T2.<br>PSA < 20 ng/mL.<br>Biopsy Gleason score of <= 6.	6,864, All GG1 at biopsy<br><br>397. All GG1 at biopsy<br><br>347. All GG1 at biopsy	<1 year<br><br>1-2 years<br><br>>2 years	1997-2007	Median: 8.3 years (6.8-10.2)<br><br>Median: 7.6 (5.7-9.7)<br><br>Median: 4.7 (3.0-7.3)	Median: 62 (58.1-65.5)<br><br>Median: 62.7 (59.7- 65.3)<br><br>Median: 62.4 (59.1-65.7)	Median: 6.7 (4.8-9.3)<br><br>Median: 6.1 (4.5-8.6)<br><br>Median: 5.4 (4.2-7.6)	cT1c: 66%<br>cT2: 33%<br><br>cT1c: 76%<br>cT2: 21%<br><br>cT1c: 82%<br>cT2:15%	Prostatectomy<br>GG1: 70%<br>CC2-3: 26%<br>GG4-5: 2%<br>GG1: 59%<br>GG2-3:34%<br>GG4-5: 3%<br>GG1: 47%<br>GG2-3: 43%<br>GG4-5: 5%				99% CSS at 7 years. No difference in immediate prostatectomy vs active surveillance	In **low-risk or intermediate-risk** patients, delays of 1-2 years and 2-7 years were associated increased risk of Gleason upgrading. No difference in EPE and PSM
**Berg 2015**	RP for PCa<br>Not undergoing active surveillance<br>No neoadjuvant therapy or radiation treatment.	Retrospective single centre cohort<br>2,212	median delay: 64 days, mean delay : 76±47	1990-2011	Histological findings at RP	Mean 60.77 (SD 7.2)	Mean 7.94 (SD 7.81)	cT1 65.4%<br>cT2 32.7%<br>cT3 1.7%	GG1: 51.2%<br>GG2-3: 39.2%<br>GG4-5: 9.6%	D'Amico<br>Low 34.9%<br>Intermediate 54.6%<br>High 10.4%	n/a	n/a	n/a	- Overall delay >75 days associated with worse pathology at RP (p=0.03)<br>- Patients with **GG1 & PSA 0-10**, delay of 150 days were associated with adverse RP pathology (p=0.038)<br>- Patients with **GG2-3 & PSA >20**, delay of 60 days were associated with adverse RP pathology (p=0.032)<br>- Patients with **GG4-5 & PSA 11-20**, delay of 30 days were associated with adverse RP pathology (p=0.041)
**Satkunasiva 2013**	PSA density <0.15<br>PSA < 15 ng/ml<br>clinical stage T1c-T2a<br>Gleason sum < 6<br><3 cores positive with no more than 50% of a core involved	Single centre retrospective cohort.<br><br>41patients on AS who went on to have RP<br><br>24 patients on AS who had GG2-3 at RP pathology<br><br>70 patients immediate RP who had GG2-3 at RP pathology	Median 35.2 months (IQR 22.8-46.6)<br><br>Median: 35.3 months (22.8-45.2)<br><br>Median: 3.0 months (2.0-3.7)	1995-2011	Median of 3.5 (IQR 2.6-4.7) years	Mean: 61.1± 5.5<br><br>Mean: 62.3±5.3<br><br>Mean: 63.1±5.1	Median: 5.5 (IQR 3.7-9.4)<br><br>Median: 5.4 (3.3-9.4)<br><br>Median: 6.5 (4.9-8.8)	cT1a 4.9%<br>cT1c 80.5%<br>cT2a 14.6%	GG 1 30%<br>GG2 45%<br>GG3 15%<br>GG4 7.5%<br>GG5 2.5%<br><br>GG1: 0%<br>GG2: 75%<br>GG3: 25%<br>GG4-5: 0%<br>GG1: 0%<br>GG2: 78.6%<br>GG3: 21.4%<br>GG4-5: 0%	NR	97.4%<br><br>94.6%<br><br>86.8%			In **low-risk** patients<br>- No difference in PSM, extraprostatic extension, pT3 disease<br>- No difference in RFS
**Abern 2013**	Not on neoadjuvant hormone or radiation treatment	Multicentre (Veterans affairs) retrospective cohort study<br><br>Total of 1561<br><br>Low risk patients:<br><3 months: (n = 510)<br>>3 and <6 months: (n = 225)<br>>6 and <9 months: (n = 45)<br>9 months: (n = 33)<br><br>Intermediate-risk patients:<br><3 months: (n = 427)<br>>3 and <6 months: (n = 250)<br>>6 and <9 months: (n = 46)<br>9 months: (n = 25)	0–90 days (0–3 months), 91–180 days<br>(3–6 months), 181–270 days (6–9 months), and 271 days or more (> 9 months)	1988-2011	Low-risk patients:<br><3 months: 68 months (35-101)<br>>3 and <6 months: 46 months (18-78)<br>>6 and <9 months: 38 months (27-59)<br>9 months: 53 (30-75)<br><br>Intermediate-risk patients:<br><3 months: 47 months (21-76)<br>>3 and <6 months: 39 months (19-67)<br>>6 and <9 months: 42 months (24-64)<br>9 months: 46 (28-50)	Low-risk patients:<br><3 months: 61 (57-66)<br>>3 and <6 months: 61 (56-64)<br>>6 and <9 months: 61 (57-64)<br>9 months: 62 (59-65)<br><br>Intermediate-risk patients:<br><3 months: 62 (58-66)<br>>3 and <6 months: 61 (57-65)<br>>6 and <9 months: 62 (58-64)<br>9 months: 62 (60-66)	Low-risk patients:<br><3 months: 5.4 (4.3–7.2)<br>>3 and <6 months: 5.3 (4.4–7.0)<br>>6 and <9 months: 5.3 (4.5–7.1)<br>9 months: (4.1–6.2)<br><br>Intermediate-risk patients:<br><3 months: 9.1 (5.3–12.5)<br>>3 and <6 months: 7.7 (5.2–11.0)<br>>6 and <9 months: 8.9 (6.1–12.2)<br>9 months: 7.8 (5.8–13.2)	Low-risk patients:<br><br><3 months: T1 72%, T2a 28%<br>>3 and <6 months: T1 76%, T2a 24%<br>>6 and <9 months: T1 78%, T2a 22%<br>9 months:<br>T1 79%, T2a 21%<br><br>Intermediate-risk patients:<br><3 months: 9.1 (5.3–12.5)<br>>3 and <6 months: 7.7 (5.2–11.0)<br>>6 and <9 months: 8.9 (6.1–12.2)<br>9 months: 7.8 (5.8–13.2)	Biopsy<br>Intermediate-risk patients:<br><3 months: GG1 34%, GG2 48%, GG3 18%<br>>3 and <6 months: GG1 25%, GG2 52%, GG3 24%<br>>6 and <9 months: GG1 37% GG2 46% GG3 17%<br>9 months:<br>GG1 32%,<br>GG2 48%,<br>GG3 20%		Intermediate risks > 9 months BCR-free survival 37& vs 70% at 5 years			For **low-risk** patients, delay of over 9 months has no effect on BCR, PSM, ECE and pathological upgrading,<br><br>But for **intermediate-risk** patients, delays > 9 months were significantly associated with BCR and PSM. also in subsets of<br>men with biopsy Gleason ≤3<br>+ 4,<br>PSA ≤6.0, and low tumour<br>Volume<br><br>Crude BCR-free survival at 5 yr in **intermediate-risk** groups and subgroups was significantly worse for >9 mo delay
**Redaniel 2013**	15+ years old<br>Surgical resection with curative intent within 6 months of diagnosis	Multicentre retrospective cohort study<br><br>17043	Median 95 days (IQR 70-125)	1996-2009	five-and ten-years, or at the end of the study period (Dec 2009)	15-54: 11.68%<br>55-64: 51.86%<br>>65: 36.46%	NR	NR	NR	NR	NR	NR		No associated between time from diagnosis to surgery and survival if definitive surgery took place within 6 months of diagnosis
**Korets 2012**	RP for PCa<br>No NHT or radiotherapy<br>More than 12 months follow-up postoperatively	Single centre retrospective study<br><br>Total 1568; 1098 ≤60<br>d, 303 61–90 d, 167 >90 d	≤60 d vs 61–90 d vs<br>>90 d	1990-2009	64 months (IQR 30, 93)	<60 days: 60 (IQR 55, 66)<br><br>60-90 days: 61 (56, 66)<br><br>>90 days: 61 (57, 66)	<60 days: 5.7 (IQR 4.3, 8.0)<br><br>60-90 days: 6.2 (4.7, 8.7)<br><br>>90 days: 6.2 (4.5, 8.2)	<60 days: T1 61.9%, T2 37.4%, T3 0.7%<br><br>60-90 days T1 61.4%, T2 36.9%, T3 1.7%<br><br>>90 days: T1 67.1%, T2 32.9%, T3 0%	<60 days:<br>GG1 50%<br>GG2+3: 37.8%<br>GG4: 12.2%<br><br>60-90 days<br><br>GG1 53%<br>GG2+3: 37.3%<br>GG4: 9.7%<br><br>>90 days<br>GG1 61.2%<br>GG2+3: 34.6%		5 years BCR-free survival of high-risk patients:<br><br><60 days: 80%, 61-90 days: 78%, >90 days: 85%<br><br>No difference; p=0.11	N/a	n/a	A delay of >60 days was not associated with adverse pathological findings at surgery and biochemical outcomes in all risks

									GG4: 4.2%					
**Sun 2012**	Age > 66 years<br>PCa as first malignant disease<br>Medicare Part A and Part B claims available<br>RP for T1-2 PCa<br>No lymphnode metastases or distant metastases<br>Low-grade tumours (Gleason sum <7)	Retrospective multicentre cohort<br><br>17153	≤3 vs 3–5 vs 5–9 vs ≥9 months<br><br>Final analysis was <3 vs >3 months	1995-2005	10 year prostate cancer mortality was reported but length of follow up not specified<br>For urinary incontinence and ED date, 18 months follow up	Median 68, mean 69 IQR 66-71	NR	NR	Gleason 2-4: 3.9%<br>Gleason 5-6: 96.1%	NR	NR	NR	No difference in cancer specific mortality at 10 years between the 2 groups	In **low-risk** patients, delay in RP (>3 months) is associated with LESS pathological upstaging, but is associated with higher rate of urinary incontinence and erectile dysfunction
**Dall-Era 2011**	No previous PCa treatment<br>Treated with AS<br>No active treatment received within 6 months of diagnosis	Retrospective case-controlled study<br><br>311 (278 immediate RP, 33 RP after AS)<br><br>Study also included additional high risks patients but with statistically significant baseline patient characteristics and biopsy between AS + RP and immediate RP group	18 months (range 7-76)	1996-2008	AS + RP: median 12 months (range <1-60)<br><br>Immediate RP: median 27 months (range <1-162)	Median 59 (range 37-79)	Median 5.8 (range 0.02->100)	AS + RP:<br>T1 64%<br>T2 36%<br><br>Immediate RP:<br><br>T1 49%<br>T2 51%	GG1 100% in both low-risk groups		Sub-group analysis of patient with >4 years f/u:<br><br>immediate RP: 98%<br><br>For AS + RP: 100%<br><br>(biochemical disease-free survival)			For both **low and high-risk** patients, AS followed by RP vs immediate RP was not associated with pathological upgrading, metastasis or positive surgical margins
**O'Brien 2011**	Low risk PCa	Retrospective cohort study (single surgeon)<br><br>Total 1111; 1052 <6 mo; 59 ≥6 mo	< 6 months vs >6 months	1989-2009	< 6 months:<br>43 months (range 1-159)<br><br>>6 months:<br>38 months (range 1-222)	< 6 months: 59 (mean)<br><br>>6 months: 61 (mean)	< 6 months: 4.7 (mean)<br><br>>6 months: 5.0 (mean)	< 6 months:<br>T1: 81.1%<br>T2:18.9%<br><br>>6 months:<br>T1: 93.2%<br>T2: 6.8%	GG 1 100%		5 year BCR extrapolated from Kaplan-Meier Curve:<br><br><6 months: 95%<br>>6 months: ~75%			In **low-risk** patients, delay of RP over 6 months was associated with greater risk of high grade disease at RP, biochemical progression, and worse progression-free survival rate
**Holmstrom 2010**	PCa for RP<br>70 years or younger at the time of diagnosis<br>T1-T2N0M0<br>GS sum < 6<br>PSA <20ng/ml<br>No hormonal treatment or treatments other than RP	Retrospective NPCR<br>2,566 men<br><br>2,344 immediate RP	Immediate RP	1997-2002	Median 8.2 years	Mean: 61.1 years (SD 5.3, range 41–70)	Mean: 7.8 (SD 3.8)	Primary<br>cT1: 61%<br>cT2: 39%	All GG 1	NR	NR	NR	At median 8.2 years follow up:<br><br>Primary RP: 99.3% disease specific survival,<br>93.8% OS	In **low-risk** patients, upgrading in Gleason score was associated with deferred RP (38% vs 35%, p<0.001)<br>No difference in PSM or EPE
		222 delayed RP	AS with deferred RP Median delay 19.2 months			Mean: 61.9 years (SD 4.5, Range 50–70)	Mean: 6.7 (SD 3.4)	Deferred<br>cT1: 81%<br>cT2: 18%	All GG 1	NR	NR	NR	Deferred RP: 99.1 % disease specific survival,<br>94.6% OS	No difference in CSS at median follow up of 8 years
**Van den Bergh 2010**	T1c/ T2 PCa<br>PSA <10 ng/ml<br>PSA density < 0.2 ng/ml<br>Gleason score < 3+3<br>1 or 2 positive cores only<br>No known lymph node or distant metastasis	Retrospective ERSPC<br>227 men<br><br>158 immediate RP	Immediate RP	1995-2009	Mean 5.2 years	Mean 62.8<br>Median 62.6	Mean 4.3<br>Median 4.0	T1c: 81.6%<br>T2: 18.4%	All GG 1	NR	91%	NR		In **low-risk** patients<br>No difference in PSM, Gleason score upgrading, tumours size<br>No significant difference RFS (p=0.185)
		69 delayed RP	AS with deferred RP:<br>Mean delay of 2.6 years		Mean: 3.2 years	Mean 62.3<br>Median 62.1	Mean 4.2<br>Median 3.8	T1c: 94.2%<br>T2: 5.8%	All GG 1	NR	78%	NR		
**Phillips 2007**	Localised PCa treated by RP or radiation therapy<br>No neoadjuvant or adjuvant therapies<br>Patient with longer than 1 year follow-up	Retrospective<br>393 men<br><br>310 immediate treatment (67% RP, 32% RT)	Immediate treatment (<3 months)	1991-2004	Median follow up 2.3 years	Median age 63.1y	Median PSA 6.5	Low T1/T2a: 91%<br>High >T2b: (%	GG 1: 71%<br>GG 2-3: 25%<br>GG 4-5: 4%					In **mixed risk** patients<br>No difference n RFS between immediate and delayed treatment (p=0.28)
		83 delayed treatment (42% RP, 58% RT)	Delayed treatment (>3 months)			Study does not differentiate age for subgroups	Study does not differentiate PSA for subgroups	Low T1/T2a: 92%<br>High >T2b: 8%	GG1: 84%<br>GG 2-3: 15%<br>GG 4-5: 1%					
**Vickers 2006**	No prior radiation therapy or neoadjuvant hormonal therapy<br>Surgery within 12 months of surgery	Retrospective<br>3149 men<br><br>Surgery <90d after bx<br>2258	Surgery <90d after bx	1987-2002	10 years	<90d<br>61 (56-65)	<90d<br>6.6 (4.7-10)	<90d<br>cT1: 40%<br>cT2: 57%<br>cT3: 3%	<90d<br>GG1: 67%<br>GG2-3: 27%<br>GG4-5: 6%	NR				In **mixed risk** patients<br>No effect of treatment delay on BCR at 3,5,8,10 year follow up
		Surgery >90d after bx<br>891	Surgery >90d after bx			>90d<br>61 (56-65)	>90d<br>5.9 (4.5-8.6)	>90d<br>cT1: 55%<br>cT2: 44%<br>cT3: 2%	>90d<br>GG1: 76%<br>GG2-3: 21%<br>GG4-5: 3%					
		150 Immediate												
**Lee 2006**	Biopsied confirmed PCa for RP<br>No concurrent sacral nerve grafting or other surgery	Retrospective<br>169 patients<br>One group	Interval between biopsy and RP	2001-2004	6 months	Mean age 59	Mean PSA 7.2	NR	NR					In **mixed risk** patients<br>Biopsy/treatment interval did not predict outcomes
**Graefen 2005**	Localised PCa for RP<br>No neoadjuvant hormonal therapy	Retrospective cohort<br>795 men		1992-2000	Mean 33 months	NR	0-4: 9.8%<br>4.1-10: 47.6%<br>10.1-20:26.7%<br>>20: 14.8%	T1-T2a:67%<br>>T2b: 33%	GG1: 60%<br>GG2-3: 36.7%<br>GG4-5: 3.7		No difference<br>p=0.8			Delay not significantly associated with BCR, even in **high grade** disease
**Khan 2004**	T1c, T2 or T3a PCa for retropubic RP	Retrospective<br>926 men		1989-1994	Mean (range) by delay period	Mean (range) by delay period	Mean (range) by delay period	60 days or less:<br>T1c: 57<br>T2a: 56	GS<br>60 days or less<br>2-6: 106		10 years BCRFS rate<br><br>60 days or less: 78%			Delay of several months does not impact long-term biochemical cancer

	No neoadjuvant or adjuvant radiation or hormone therapy	60 days or less: 162<br>61-90 days: 268<br>91-120 days: 247<br>121-150 days: 130<br>151+ days: 119			60 days or less: 8 (1-14)<br>61-90 days: 8.2 (1-14)<br>91-120 days: 8.5 (1-14)<br>121-150 days: 7.6 years (1-14)<br>151+ days: 7.8(1-13)	60 days or less: 57 (39-73)<br>61-90 days: 58 (42-72)<br>91-120 days: 59 (41-75)<br>121-150 days: 59 (39-71)<br>151+ days: 60(39-71)	60 days or less: 9.4 (0.4-41.8)<br>61-90 days: 8.6 (0.4-60)<br>91-120 days: 8.4 (0.4-70.3)<br>121-150 days: 8.5 (0.4-66)<br>151+ days: 7.1 (0.5-28.8)	T2b: 34<br>T2c: 7<br>T3a: 8<br><br>61-90 days:<br>T1c: 94<br>T2a: 92<br>T2b: 52<br>T2c: 18<br>T3a: 12<br><br>91-120<br>T1c: 89<br>T2a: 91<br>T2b: 43<br>T2c: 13<br>T3a: 11<br><br>121-150<br>T1c: 62<br>T2a: 39<br>T2c: 7<br>T3a: 3<br><br>151+<br>T1c: 68<br>T2a: 34<br>T2b: 10<br>T2c: 6<br>T3a: 1	7: 48<br>8-10: 8<br><br>61-90 days:<br>2-6: 187<br>7: 66<br>8-10: 15<br><br>91-120<br>2-6: 180<br>7: 55<br>8-10: 12<br><br>121-150<br>2-6: 95<br>7: 29<br>8-10: 6<br><br>151+<br>2-6: 101<br>7: 16<br>8-10: 2		61-90 days: 78%<br>91-120 days: 75%<br>121-150: 82%<br>151+ days: 87%			control rates in risk stratified patients<br><br>Delay of surgery have significant adverse effects on positive lymph node and positive seminal vesicle rates
**Nam 2003**	RP for PCa<br>No evidence of residual disease<br>PSA > 0.1 postoperatively<br>No lymph node involvement<br>No neoadjuvant therapy	Retrospective<br>645<br><br><3mo: 456<br>>3mo: 189	<60 d vs >60d	1987-1997	10 years	Mean (rage): 62.6 (39.2-74.5)	<4.0: 111<br>4.1-10: 279<br>10.1-20: 135<br>>20.0: 60	T1: 297<br>T2: 348	GS<br>2-6: 229<br>7: 350<br>8-10: 66		10-year BCRFS:<br>No delay: 74.6%<br>Delay: 61.3%			Risk of biochemical recurrence and metastasis is significantly higher in those with delay in RP
**Hirasawa 2017**	RP for T1-T3N0M0 PCa<br>No Neoadjuvant hormone treatment or radiation therapy<br>Postoperative follow-up period > 6 months<br>Less than 12 months from diagnosis to treatment	793 patients<br>Three groups:<br>1. <3m -196 pts<br><br>2. 3-6m – 513 pts<br><br>3. >6m – 84 pts	<3m to >6m	2017	32.2 months (median)<br>IQR 6.1 – 102.3 months	Overall 64.4+/- 6.4<br>1. <3m = 64.1 +/- 6.4<br><br>2. 3-6m = 64.5 +/- 6.5<br><br>3. >6M = 64.3 +/- 6.3	Overall 9 +/- 6.3<br>1. <3m=9.4 +/- 6.2<br><br>2. 3-6m=8.7 +/- 6.4<br><br>3. >6m=9.9 +/- 6.0	cT1: 576 (72.6%)<br>cT2a: 135 (17%)<br>cT2b: 50 (6.3)<br>cT2c: 29 (3.7%)<br>cT3ab: 3 (0.38%)	GG1: 192 (24.2%)<br>GG2-3: 425 (53.6%)<br>GG4-5: 176 (22.2%)	NR	No difference in 5 year BCRFS between all three groups:<br>1 – 76%<br>2 – 80.7%<br>3 – 82.6%<br>P=0.99			Delay did not affect recurrence in both **mixed risks** and **high-risk patients**
**Tosoian 2016**	Very-low risk<br>Clinical stage T1c disease<br>PSA density <0.15 ng/ml<br>GS 6<br>Two or fewer positive biopsy cores, and 50% involvement of any core with cancer.<br>Low risk:<br>clinical stage T2a<br>PSA <10 ng/ml<br>GS 6	Total = 3877 pts<br><br>Delayed = 89<br><br>Immediate = 3788	Delayed = median of 2 years after diagnosis	2004 – 2014	5 year (range 0.01 – 18.0 years)	Del Median: 65 (range 55-83)<br><br>Imm Median: 58 (36-75)	Del Median: 5.0 (0.7-17)<br><br>Imm Median: 4.7 (0.01-20)	Delayed:<br>pT2-71 (79.8%)<br>pT3a+b-18 (21.2%)<br><br>Immediate<br>pT2-3182 (84%)<br>pT3a+b-606 (16%)	Delayed<br>GG1: 48 (54%)<br>GG2-3: 37 (31.8%)<br>GG4-5: 4 (4.5%)<br><br>Immediate<br>GG1: 2410 (63.6%)<br>GG2-3: 1256 (33.2%)<br>GG4-5: 122 (3.2%)	NR	Median follow up was 2 years: no difference in time to BCR between two groups<br>Del BCR 6 (6.7%)<br>Imm BCR 160 (4.2)<br>P=0.1			**In low-risk patients,** decision to initiate AS of favourable-risk PCa is not independently associated with adverse pathologic findings
**Rodríguez Alonso 2009**<br><br>**Abstract only available**	PCa with at least two postoperative determinations of PSA.<br>No neoadjuvant therapy or immediate adjuvant therapy	232 patients	Delay defined as >6 months from biopsy to operation	2000 – 2007	Mean (SD)<br>< 6 months: 38.62 (20.26)<br>> 6 months: 32.45 (22.84)	Mean (SD):<br>Organ confined: 65.4 (5.99)<br>Extracapsular: 65.84(5.12)	Mean (SD):<br>Organ confined: 8.6 (4.56)<br>Extracapsular: 7.94 (3.53)	NR	GS<br>Mean (SD):<br>Organ confined: 5.69 (1.16)<br>Extracapsular: 6.09 (1.23)	NR in abstract	BRFS in delay </=6mo:<br>86.1, 78.4, 78.4 78.4% at 1,2,5,7 years<br>>6m:88.9, 82.8, 77.4, 77.4, 77.4 at 1,2,5,7 years<br>P=0.632 – delay not a/w BRFS			Reasonable surgical delay does not cause significant negative impact
**Freedland 2006**	RP for low risk PCa<br>PSA < 10<br>Gleason sum <6	895 men total<br><br><90 days = 672<br><br>91 – 180 days = 175<br><br>>180 days 48		1988-2004		Mean age +/- SD<br><90 = 61.5 +/- 6.7<br><br>91 – 180<br>61.6 +/- 7.2<br><br>>180<br>60.3 +/- 6.9	Median PSA<br><90 = 5.4<br><br>91 – 180 = 5.8<br><br>>180 = 5.6	<90<br>cT1 = 329<br>cT2x = 13<br>cT2a = 211<br>cT2b = 64<br>cT2c/cT3 = 36<br>90 – 180<br>cT1 = 91<br>cT2x = 2<br>cT2a = 45<br>cT2b = 25<br>cT2c/cT3 = 8<br>91 – 180<br>cT1 = 31<br>cT2x = 2<br>cT2a = 8<br>cT2b = 5<br>cT2c/cT3 = 1	<90<br>GG1 = 475<br>GG 2 = 136<br>GG 3-5 = 46<br><br>90 – 180<br>GG1 = 118<br>GG 2 = 40<br>GG 3-5 = 14<br><br>>180<br>GG1 = 33<br>GG 2 = 12<br>GG 3-5 = 3		15 year actuarial prostate cancer specific survival rates after BCR in patients with recurrence at 3 years or less is 41% vs 87% in patients with recurrence more than 3 years after prostatectomy			>180 days delay associated with significantly worsened PSA free survival in **low-risk** patients
**Boorjian 2005**	RP for localised PCa<br>No previous cancer therapy in the form of radiation or hormones<br>RP within 1 year of diagnosis	3149 pts<br>RP <90d after bx=2258<br><br>RP>90d=891	>90 days	1987-2002	5.4 (2.2-7.9) years after RP	61 (56-65)	<90d: 6.6 (4.7-10.0)<br><br>>90d: 5.9 (4.5-8.6)	<90d:<br>cT1: 885 (40%)<br>cT2: 1288 (57%)<br>cT3: 67 (3%)<br>>90d:<br>cT1: 483 (55%)<br>cT2: 391 (44%)<br>cT3: 14 (1%)	Not defined	Not defined	No greater risk of BCR if >3m delay			Delay of up to a year did not correlate with probability of BCR in **high risk** and mixed risk patients

**Xia 2020**	Clinically localised high risk PCa<br>CT1-2cN0cM0<br>Not any form of neoadjuvant therapies<br>Underwent lymph node dissection<br>Aged between 40 and 80<br>Delay time between 30 days and 18 days	32184	31 to 60 days<br>61-90 days<br>91-120 days<br>121-150 days<br>151-180 days	2006-2016	Median (IQR):<br>41.7 (23.3-65.4)	Median (IQR):<br>SDT<br><br>31-60 days: 64 (59-68)<br>61-90 days: 64(59-68)<br>121-150 days: 63(58-68)<br>151-180 days: 63(58-67)	No.<br><br>31-60 days:<br><br><10: 8027<br>10 to <20: 2241<br>> 20: 3536<br><br>61-90 days:<br><10: 6686<br>10 to <20: 1850<br>> 20: 3214<br><br>91- 120 days:<br><10: 2350<br>10 to <20: 735<br>> 20: 1404<br><br>121-150 days:<br><10: 702<br>10 to <20: 224<br>> 20: 578<br><br>151-180<br><10: 265<br>10 to <20: 98<br>> 20: 274		GS<br>31-60 days:<br>6: 506<br>7: 1737<br>8: 6685<br>9-10: 4876<br><br>61-90 days:<br>6: 512<br>7: 1732<br>8: 6148<br>9-10: 3358<br><br>91- 120 days:<br>6: 267<br>7: 768<br>8: 2361<br>9-10: 1093<br><br>121-150 days:<br>6: 139<br>7: 304<br>8: 759<br>9-10: 302<br><br>151-180<br>6: 63<br>7: 164<br>8: 192<br>9-10: 118	All high- and very- high-risk (PSA >20 ng/ml or Gleason 8-10 and NCCN)				Delay in surgery not associated in any adverse pathological outcomes and worsened overall survival in **very-high-risk** or **high-risk patients**

Table S3 – Risk of bias assessment of observational studies comparing delayed RP and immediate RP

	Study	Selection	Comparability	Outcome
**Aas 2018**	Is time from diagnosis to radical prostatectomy associated with oncological outcomes?	★★★	★	★★
**Abern 2013**	Delayed radical prostatectomy for intermediate-risk prostate cancer is associated with biochemical recurrence: possible implications for active surveillance from the SEARCH database.	★★★	★	★
**Anil 2018**	Impact of Delay from Biopsy to Surgery on the Rate of Adverse Pathologic and Oncologic Outcomes for Clinically Localized Prostate Cancer	★★★	★	★
**Awasthi 2019**	Optimizing Time to Treatment to Achieve Durable Biochemical Disease Control after Surgery in Prostate Cancer: A Multi-Institutional Cohort Study	★★★	★★	★
**Berg 2015**	Delay from biopsy to radical prostatectomy influences the rate of adverse pathologic outcomes	★★★	★	★
**Boorjian 2005**	Does the time from biopsy to surgery affect biochemical recurrence after radical prostatectomy?	★★★	★★	★
**Dall-Era 2011**	Surgical management after active surveillance for low-risk prostate cancer: Pathological outcomes compared with men undergoing immediate treatment.	★★★	★★	★★

**Filippou 2015**	Immediate Versus Delayed Radical Prostatectomy: Updated Outcomes Following Active Surveillance of Prostate Cancer	★★★	★★	★★
**Fossati 2017**	Evaluating the effect of time from prostate cancer diagnosis to radical prostatectomy on cancer control: Can surgery be postponed safely?	★★★	★★	★
**Freedland 2006**	Delay of radical prostatectomy and risk of biochemical progression in men with low risk prostate cancer.	★★★	★	★
**Graefen 2005**	Reasonable delay of surgical treatment in men with localized prostate cancer—impact on prognosis?	★★★	★★	★★
**Gupta 2019**	Evaluating the Impact of Length of Time from Diagnosis to Surgery in Patients with Unfavorable Intermediate to Very High-Risk Clinically Localized Prostate Cancer	★★★	★	★
**Hirasawa 2017**	No clinical significance of the time interval between biopsy and robotic-assisted radical prostatectomy for patients with clinically localized prostate cancer on biochemical recurrence: a propensity score matching analysis.	★★★	★	★★
**Holmstrom 2010**	Outcome of primary versus deferred radical prostatectomy in the national prostate cancer register of Sweden follow-up study.	★★	★	★
**Khan 2004**	Impact of surgical delay on long-term cancer control for clinically localized prostate cancer.	★★★	★	★★★

**Korets 2012**	Effect of delaying surgery on radical prostatectomy outcomes: a contemporary analysis	★★★	★★	★
**Lee 2006**	Does the interval between prostate biopsy and radical prostatectomy affect the immediate postoperative outcome?	★★★	★★	★
**Loeb 016**	Immediate versus delayed prostatectomy: Nationwide population-based study.	★★★	★★	★★
**Nam 2003**	Delay in surgical therapy for clinically localized prostate cancer and biochemical recurrence after radical prostatectomy.	★★★	★★	★★
**O'Brien 2011**	Delay of surgery in men with low risk prostate cancer.	★★★	★★	★
**O'Callaghan 2017**	Prostate cancer outcomes and delays in care	★★★	★★	★
**Patel 2019**	The effect of time from biopsy to radical prostatectomy on adverse pathologic outcomes	★★★	★	★
**Phillips 2007**	Does a delay in initiating definitive therapy affect biochemical recurrence rates in men with clinically localized prostate cancer?	★★★	★★	★
**Redaniel 2013**	Time from diagnosis to surgery and prostate cancer survival: a retrospective cohort study	★★★	★★	★★
**Rodríguez Alonso 2009**	Impact of surgical delay on pathological findings and prognosis of patients with prostate cancer.	★★★	★★	★
**Satkunasivam 2013**	Pathological, oncologic and functional outcomes of radical prostatectomy following active surveillance.	★★★	★	★

**Sun 2012**	Is a treatment delay in radical prostatectomy safe in individuals with low-risk prostate cancer?	★★★	★	★
**Tosoian 2016**	Pathologic Outcomes in Favorable-risk Prostate Cancer: Comparative Analysis of Men Electing Active Surveillance and Immediate Surgery.	★★	★★	★
**Van den Bergh 2010**	Is delayed radical prostatectomy in men with low-risk screen-detected prostate cancer associated with a higher risk of unfavorable outcomes?	★★★	★★	★
**Vickers 2006**	Does a delay between diagnosis and radical prostatectomy increase the risk of disease recurrence?	★★★	★★	★
**Westerman 2018**	Impact of time from biopsy to surgery on complications, functional and oncologic outcomes following radical prostatectomy	★★★	★★	★★
**Xia 2020**	Surgical Delay and Athological Outcomes for Clinically Localized High-Risk Prostate Cancer	★★★	★★	★
**Zanaty 2017**	Does surgical delay for radical prostatectomy affect patient pathological outcome? A retrospective analysis from a Canadian cohort	★★★	★★	★

Table S4 – A summary of baseline characteristics and outcomes of included RCTs comparing NHT and immediate RP

Study	Inclusion and Exclusion Criteria	Year of treatment	Average duration of follow-up	No. of patients (NHT+RP / RP Only)	Average Age	Average PSA	Tumour Characteristics		Comparison	Outcome
**Ahlgren 1999<br>Hugosson 1999<br>AUS 2002**	T1b -T3aNXM0 PCa<br>Age < 75 years<br>Life expectancy of > 10 years<br>No previous PCa treatment	1991-1994	Median (range) of 82 (8-104) months	63/63	Mean (range)<br>NHT +RP<br>67 (50-77) years<br><br>RP Only 66(54-77) years	Mean (range) PSA (ng/ml)<br>NHT: 12.0 (1.5-210.0)<br>RP: 11.2 (0.8-130.0)	RRP<br>T1-T2a: 20<br>>T2b: 43<br>GS 2-4 : 1<br>GS 5-6 : 22<br>GS 7-10 : 40	NHT<br>T1-T2a: 25<br>>T2b: 38<br>GS 2-4 : 2<br>GS 5-6 : 26<br>GS 7-10 : 35	triptorelin 3.75 mg IM monthly for 3-months cyproterone acetate 50mg BD (1 week before and 2 weeks after first injection) + RP vs RP only	No significance difference between OS, PFS, Mets, CSS, PSA Failure (Two consecutive PSA values >0.5 ng/ml)<br>Significantly less PSM in NHT + RP group
**Dalkin 1996**	Localised PCa localised<br>Life-expectancy > 10 years<br>Patients elected to have radical prostatectomy<br>PSA > 4.0 ng/mL Stage T1C, T2A or T2B<br>Bone scan to excluded bone metastasis	NR	No long-term follow-up	28/29	Mean age (range)<br><br>NHT +RP:<br>65.6 (50-76)<br><br>RP:<br>64.7 (48-76)	NHT+RP<br>4.1 – 10: 16<br><br>10.1- 20: 9<br><br>>20: 3<br><br>RP only:<br>4.1 – 10 = 18<br><br>10.1- 20 = 9<br><br>>20 = 1	T stage<br>NHT + RP<br>T1c =17<br>T2a = 8<br>T2b = 3<br><br>RP only<br>T1c = 16<br>T2a = 12<br>T2b = 0	GS<br>NHT + RP<br>2-4 = 8<br>5-7 = 16<br>8-10 = 4<br><br>RP only<br>2-4 = 6<br>5-7 = 21<br>8-10 =1	Goserelin acetate 3.6ng subcutaneous monthly for 3-months vs immediate retropubic RP	No difference in organ confinement and positive lymph node involvement found between two groups
**Fair 1997**	Clinical T1-T2 PCa<br>Good performance status<br>No prior hormonal or radiation therapy	NR	NR	66/65	NR	NR	NR		Gosrelin 3-months	Rates of PSM and Organ confinement is significantly better in patients undergoing NHT+RP in respect to RP only
**Gravina 2007**	Histologicaly proven, clinical stage T2-T3a PCa<br><br>No previous hormonal therapy, radiotherapy or chemotherapy or investigational agents.<br><br>Life expectancy > 10 years	2002-2003	No long-term follow-up	61/58	Median (range):<br><br>NHT: 69(51-75)<br><br>RP Only: 67(34-76)	Median (range):<br><br>NHT: 9.6(4.7-12.4) ng/ml<br><br>RP: 8.9 (5.1-13.9) ng/ml	Clinical Stage<br>NHT:<br>cT2: 15<br>cT3a: 46<br><br>RP:<br>cT2: 22<br>cT3a: 36	Gleason Score<br>NHT:<br>≤6: 36<br>7: 19<br>8-10: 6<br><br>RP:<br>≤6: 30<br>7: 26<br>8-10: 2	Bicalutamide 3-months	Rates of PSM is significantly lower in patients undergoing NHT+RP in respect to RP only
**Klotz 2003<br>Goldenberg 1996<br>Rabbani 1998**	Previously untreated histologically confirmed, localised PCa (T1 or T2)<br>Negative bone scan Enzymatic prostatic acid phosphatase (< 1.8 units per L.)<br>PSA less than 50 ng/ml<br>No hepatic disease, renal dysfunction, and history of other malignancy within the last 5 years.	1993-1994	Mean (range): 71 (7-117)	112/101	Median age<br><br>NHT + RP: 63<br><br>RP Only: 64	NHT+RP<br>Less than 10ng/ml: 61<br><br>10-20 ng/ml: 26<br><br>Greater than 20: 18	NHT+RP<br><br>GS 2-6: 75<br>GS 7: 21<br>GS 8-10: 8<br>T1b/c: 12<br>T2a: 36<br>T2b: 19<br>T2c: 41	RP Only<br><br>GS 2-6: 73<br>GS 7: 17<br>GS 8-10: 8<br>T1b/c: 7<br>T2a: 35<br>T2b: 21<br>T2c: 32	Cyproterone Acetate 300mg O.D for 3-months + RP vs. RP Only	No significant in between PSA failure (Two consecutive measurements <0.2ng/ml), OS, CSS PSM, lymph nodes involvement, Organ confinement is significantly better in those given NHT<br>Seminal vesicle involvement is significantly worse in those given NHT
**Labrie 1997<br>Labrie 1994<br>Vaillancourt 1996**	Aged 46-72<br>Histologically proven PCa<br>Predicted life expectancy of > 10 years	From 1988	No long-term follow-up	90/71	Mean (SEM) age<br><br>NHT + RP:<br>62.5 (0.5)<br><br>RP only:<br>62.7 (0.6)	Average (range):<br><br>NHT +RP: 27 (1-53) ng/ml<br><br>RP only:<br>19 (1-37) ng/ml	NHT + RP:<br>B1: 23<br>B2: 16<br>C1: 2<br>C2: 4	RP:<br>B1: 30<br>B2: 11<br>C1: 5<br>C2: 3	Leuprolide acetate IM 7.5mg for 3-months or subcutaneous 250µg for 3-months<br>Flutamide PO 250mg t.d.s for 3-months + RP or RP only	PSM, downstaging, upstaging and organ confinement is significantly improved in patients undergoing NHT

**Prezioso 2004**	Histopathologically proven PCa<br>Stage A1 = T1a (T1a/N0/M0), A2 = T1b (T1b/N0/M0) = T1c (T1c/N0/M0), B1 = T2a (T2a/N0/M0), or B2 = T2b (T2b/N0/M0),<br>Life expectancy of < 5 years<br>WHO performance status up to 2<br>Not been previously treated with NHT or Chemotherapy.<br>Not had previous orchiectomy<br>Not had previous neoplasia<br>Not affected by other serious comorbidities	NR	6-months	81/86	Mean age<br>NHT+ RP: 64.9<br><br>RP: 64.5	NHT + RP<br><br>Median (range): 10.1 (0.003-96.72	NR	Leuprolide acetate IM 3.75mg monthly 3-months<br>Cyproterone acetate 300 mg IM weekly (once a week for 3 weeks starting 1 week before the first leuprolide injection) + RP or RP only	PSM and lymph node involvement is significantly improved in those undergoing NHT
**Schulman 2000**<br>**Witjes 1997**	Newly diagnosed histological confirmed T2–3NxM0 PCa<br>PSA <100 ng/ml	1991-1995	4-years	192/210	NR	Mean (SD)<br><br>NHT + RP: 19.9 (17.5)	NR	Goserelin 3.6mg subcutaneous depot monthly for 3-months<br>Flutamind 250mg t.d.s for 3-months vs. direct radical retropubic prostatectomy	No difference is PSA failure (Two consecutive measurements >1 ng/ml), CSS<br><br>PSM, Organ confinement, downstaging and positive lymph nodes is significantly better in patients undergoing NHT
**Selli 2002**<br>**Bono 2001**<br>**Montironi 1999**<br>**Pagano 1998**	patients with surgically resectable clinical stage B or C (T2–T3, N0, M0) PCa	1996 -2000	No long-term follow-up	143 (3-months)/ 122 (6-months)/ 128	Mean (range)<br><br>NHT (3-months) + RP: 65.43 (49-76)<br><br>NHT (6-months) + RP:<br>66.16 (51-76)<br><br>RP: 65.72 (52-76)	Median (range)<br><br>NHT (3-months) + RP:<br>10.15 (0.0-131.8)<br><br>NHT (6-months) +RP:<br>10.0 (1.4-100.3)<br><br>RP only:<br>10.20 (0.8-763.6)	Clinical Stage<br>NHT (3-months) + RP<br>B: 108<br>C: 35<br><br>NHT (6-months) + RP<br>B: 91<br>C: 31<br><br>RP only<br>B: 99<br>C: 29	Goserelin IM 3.5mg subcutaneously 3-months or 6-months<br>Bicalutamide 50mg PO O.D. 3-months or 6-months + RP or immediate RP only	PSM, Upstage, Downstage is significantly better in patients who underwent 3-months or 6-months NHT compared to RP only
**Soloway 1995**<br>**Soloway 2002**	cT2bNxM0 histologically confirmed PCa<br>Normal radionuclide bone scan<br>PSA < 50 ng./ml<br>Age <75 years<br>No contraindication to NHT<br>Not received any hormonal therapy or chemotherapy previously	1992-1994	5-years	136/144	Mean age<br><br>NHT+ RP: 64.9<br><br>RP: 65.4	Mean (range):<br><br>NHT +RP: 14.3 (0.6-50.3)<br><br>RP: 12.5 (0.7-54.8)	Mean GS<br>NHT+ RP: 6.1<br>RP: 5.8	Cyproterone acetate 300mg O.D. for 3-months + RP vs RP (retropubic or perineal) and modified bilateral lymphadenectomy	PSM is significantly less likely in patients undergoing NHT.<br>Seminal vesicle involvement, positive lymph nodes and PSA failure (two consecutive measures > 0.4 ng/ml) does not differ between the two groups
**Yee 2010**	Localised PCa<br>No previous hormonal radiotherapy or chemotherapy	1992- 1996	Median (IQR):<br>8.0 (5.2-10.7) years	72/64	Median (IQR)<br><br>NHT + RP: 61 (57-66)<br><br>RP: 61 (57-65)	NHT + RP:<br><br><10 = 45<br>10-20 = 19<br>>20 = 8<br><br>RP: <10 = 44<br>10-20 = 15<br>>20 = 5	NHT + RP:<br>T1b/c = 30<br>T2a = 12<br>T2b =25<br>T2c = 5<br>T3 = 0<br><br>GS 2-6 = 34<br>GS 7 = 23<br>GS 8-10 = 15<br><br>RP:<br>T1b/c = 27<br>T2a = 8<br>T2b =20<br>T2c = 8<br>T3 = 1<br><br>GS 2-6 = 33<br>GS 7 = 10<br>GS 8-10 = 21	Goserelin acetate 3.6mg subcutaneous monthly 3-months<br>Flutamide 250mg TD 3-months + RP vs RP only	PSM is significantly improved in patients undergoing NHT<br><br>No differences between organ confinement, OS, CSS, lymph node involvement and seminal vesical involvement were found

Figure S7 – Risk of bias of RCTs comparing NHT and immediate RP

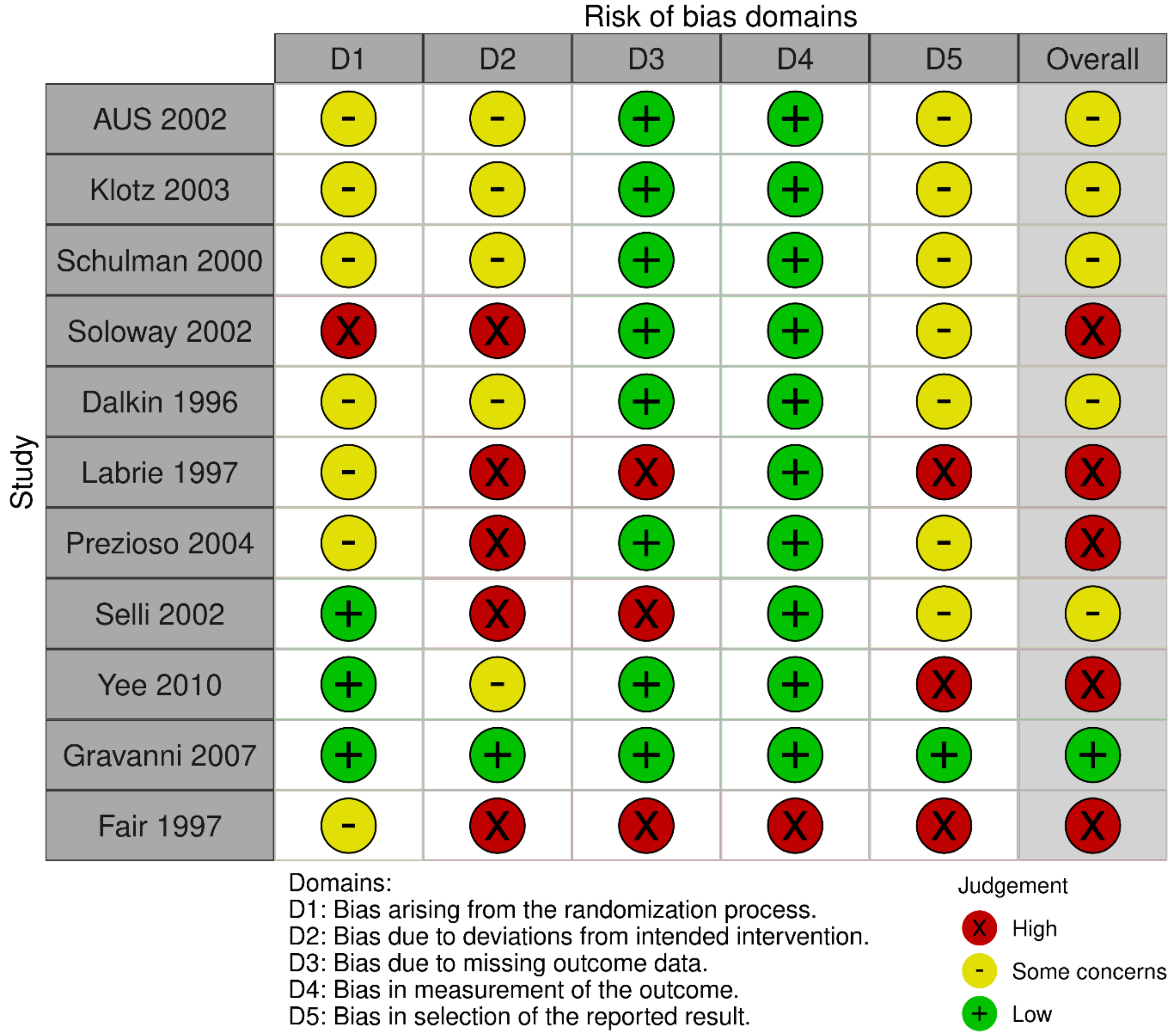

(a) Risk of bias of individual studies

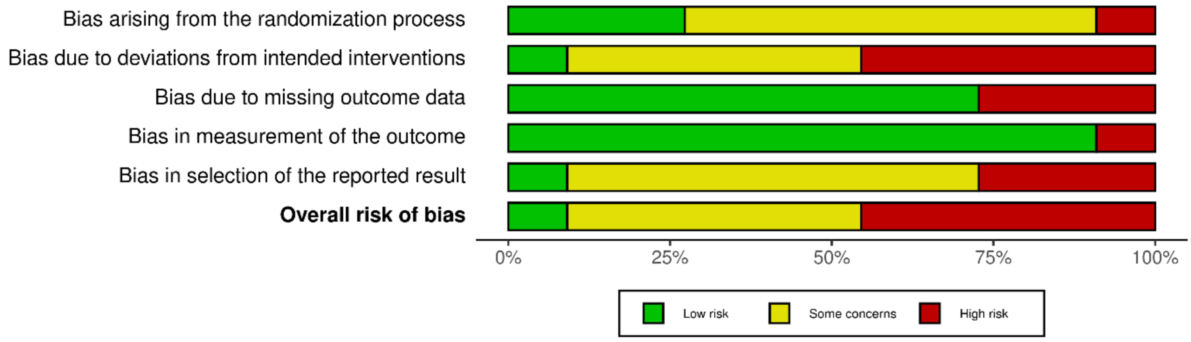

(b) Summary of risk of bias of all included studies

Figure S8 – Overall deaths in patients undergoing 3-months NHT followed by RP and immediate RP

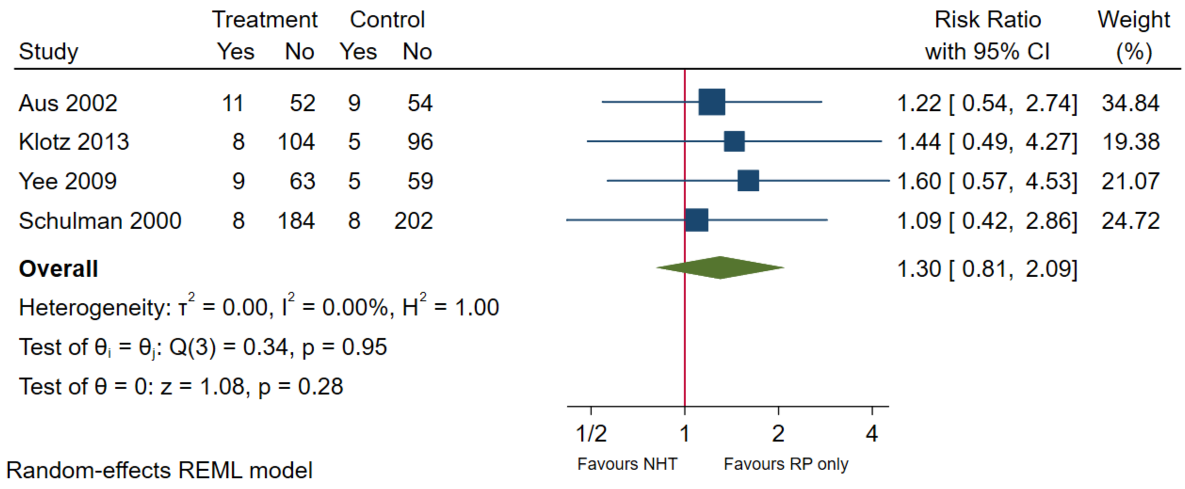

Figure S9 – Cancer-specific deaths in patients undergoing 3-months NHT followed by RP and immediate RP

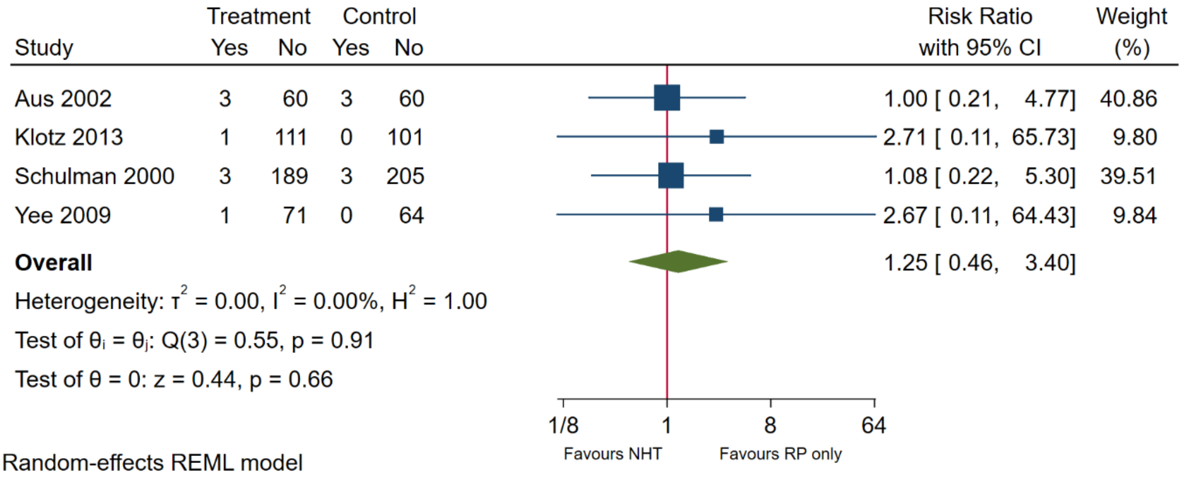

Figure S10 – seminal vesicle involvement in patients undergoing 3-months NHT followed by RP and immediate RP

Study	Treatment Yes	Treatment No	Control Yes	Control No	Risk Ratio with 95% CI	Weight (%)
Soloway 2002	20	118	30	104	0.65 [0.39, 1.08]	41.30
Goldenberg 1996	29	72	13	78	2.01 [1.11, 3.62]	39.30
Yee 2009	3	69	4	60	0.67 [0.16, 2.87]	19.41
**Overall**					1.02 [0.44, 2.32]	

Heterogeneity: $\tau^2 = 0.36$, $I^2 = 73.20\%$, $H^2 = 3.73$

Test of $\theta_i = \theta_j$: Q(2) = 8.42, p = 0.01

Test of $\theta = 0$: z = 0.04, p = 0.97

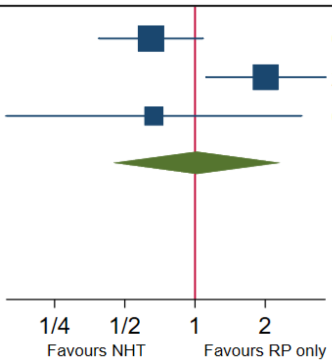

Random-effects REML model

Figure S11 – pathological upstaging in patients undergoing 3-months NHT followed by RP and immediate RP

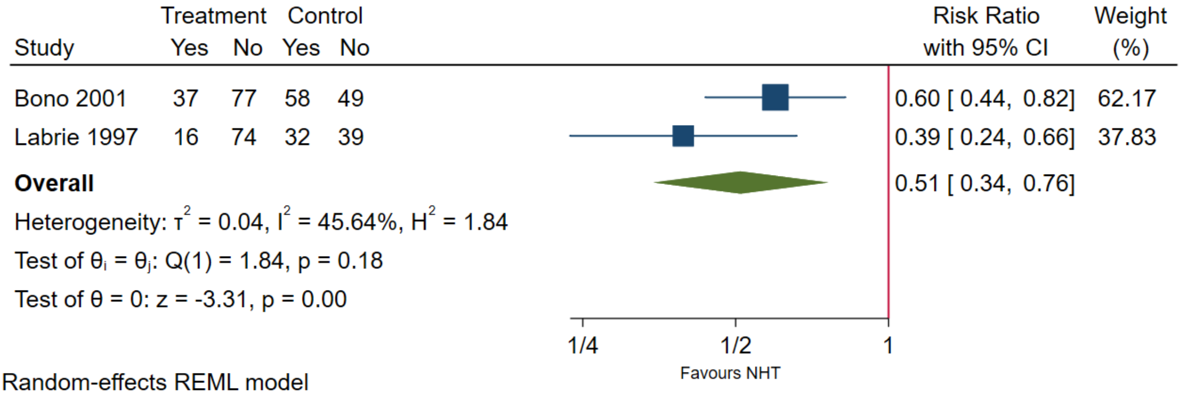

Figure S12 – pathological downstaging involvement in patients undergoing 3-months NHT followed by RP and immediate RP

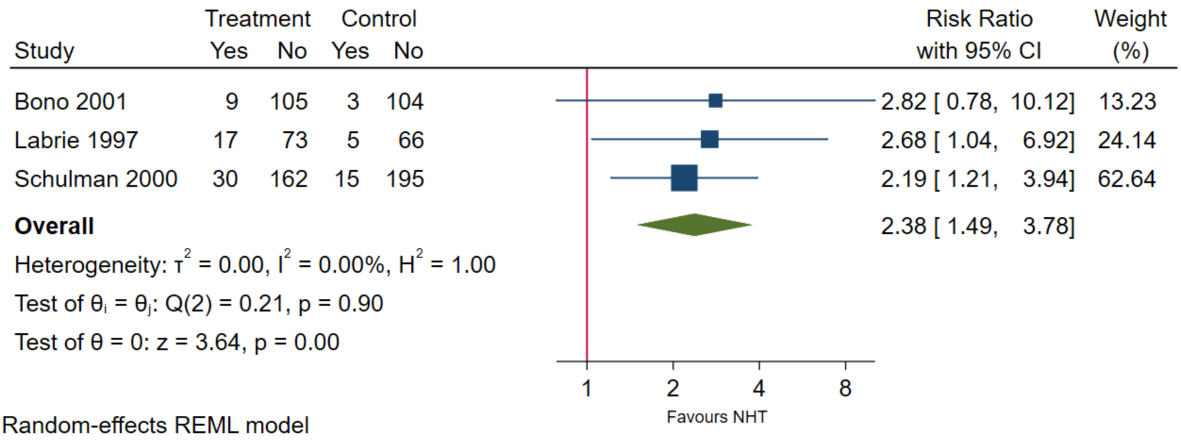

Figure S13 – PSA failure involvement in patients undergoing 3-months NHT followed by RP and immediate RP

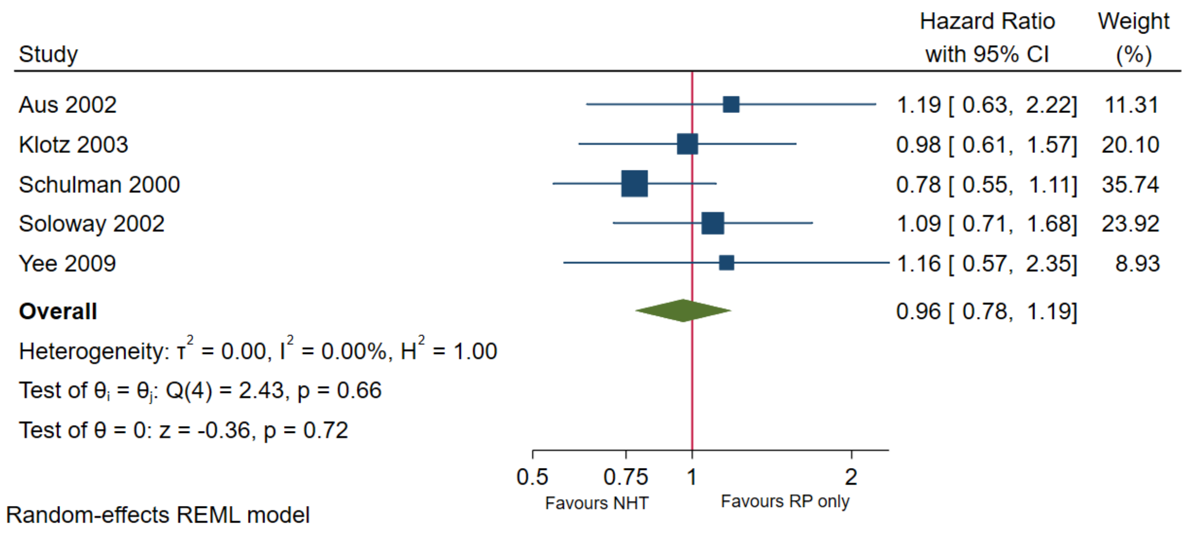

Table S5 – A summary of baseline characteristics and outcomes of non-randomised studies comparing NHT and immediate RP

Study	Study design / number of patients	Definition of delayed surgery / duration of delay	Year of treatment	Median (IQR) / mean (range) follow-up	Median (IQR)/ mean (range) age	Median (IQR)/ mean (range) PSA	cT stage	GG score	Disease risk	RFS	PFS	CSS / OS	Outcome
**Fujita 2017**	613 patients	6 months	1996 to 2016	62.9 months (IQR 34.5–102 months)<br>NCHT<br>49.8 (28.4 -77.8)<br>RP<br>111 (83.7 -138)	NCHT<br>68 (64-72)<br>RP<br>68 (64-71)	NCHT<br>9.94 (6.19-21.2)<br>RP<br>11.8 (6.45 – 24.1)	NCHT<br>1c 28.7%<br>2a 12.4<br>2b 6.2<br>2c 11.7<br>3 39.4<br>4 1.7<br>RP<br>1c 38.4<br>2a 19.2<br>2b 4.0<br>2c 8.5<br>3 30.5<br>4 1.1	NCHT<br>6 2.1%<br>7 21.6<br>>= 8 76.4<br>RP<br>6 7.3<br>7 36.7<br>>= 8 55.4	NCHT<br>1 57.1%<br>2 28.9<br>3 14.0<br>RP<br>1 79.7%<br>2 14.1<br>3 6.2	10 yr BRFreeS rates<br>NCHT 79.2%<br>RP<br>56.1%	Not available	5 yr – 98.5%<br>10 yr – 92.6%<br>The 10-year OS rate in the EMP group was significantly higher compared to the non-EMP group (*P* = 0.021).	RP with neo-adjuvant CHT using EMP for high-risk PCa patients provided excellent long-term OS
**Hsu 2007**	235 patients	6–12 weeks	1987 to 2004	74.7 (7–184) months<br>RP<br>70.6 (7–177)<br>NHT<br>98.0 (12–184)	RP<br>63.3 (41-79)<br>NHT<br>65.1 (51-75)	RP<br>14.9 (1.0 – 127.0)<br>NHT<br>18.0 (1.1 – 184.6)	Not available	RP<br><=6 22%<br>7 38<br>>= 7 41<br>NHT<br><=6 14%<br>7 43<br>>= 7 43	Not available	BFS<br>Total<br>56.7%<br>RP 59.5<br>NHT 43.4	CPFS<br>Total<br>92.9%<br>RP 95.9<br>NHT 77.6	CSS<br>Total<br>97%<br>RP 98.1<br>NHT 88.7<br>OS<br>Total<br>93.8%<br>RP 95.9<br>NHT 79.8	nADT can decrease tumour size but does not reduce the positive surgical margin rate, nor improve the survival rate in unilateral cT3a disease
**Kim 2018**	Propensity score matching<br>176 patients	Median treatment duration 4 months	2004 to 2015	49.1 (7.1-148.3) months	RP<br><65 34%<br>>= 65 66%<br>NHT<br><65 42%<br>>= 65 58%	RP<br><40 74%<br>>= 40 26%<br>NHT<br><40 74%<br>>= 40 26%	RP<br>2 2%<br>>= 3 98%<br>NHT<br>2 2%<br>>= 3 98%	RP<br><7 18<br>>= 7 82<br>NHT<br><7 12<br>>= 7 88	High risk and locally advanced	42 (range, 1-48) months<br>NHT vs RP<br>HR (95%CI)<br>0.84 (0.45-1.49) p 0.554	Not available	Not yet achieved	NHT not significant factor for predicting RM positivity, BCR free survival, OS with locally advanced or high-risk prostate Ca
**McClintock 2019**	IPTW (inverse probability of treatment weighting approach)<br>National cancer database – 386,027 patients	Not available	2005 to 2014	NHT 65.28 months<br>RP 56.90 months	RP<br><65 67.06%<br>>= 65 32.94%<br>NHT<br><65 63.88%<br>>= 65 36.17%	RP<br><10 77.39<br>10-20: 10.01<br>>20 5.30<br>unknown 7.30<br>NHT<br><10 74.99<br>10-20 11.10<br>>20 6.01<br>unknown 7.91	RP<br>1 68.55<br>2 28.76<br>3 2.62<br>4 0.1<br>NHT<br>1 68.72<br>2 28.16<br>3 3.03<br>4 0.1	Not available	RP<br>Low 28.06<br>Int 54.48<br>High 16.13<br>Very high 1.33<br>NHT<br>Low 25.30<br>Int 55.01<br>High 18.11<br>Very high 1.57	Not available	Not available	Not available	Increased utilisation in NHT in recent years, NHT decreased risk of positive surgical margins in low and intermediate risk groups
**Naiki 2012**	342 patients	3.8 (0.5-24) months	2004 to 2009	4.5 (2.0-7.5) years	RP<br>66.3 +/-6.1<br>NHT<br>67.7 +/-5.4<br>NHT <3 months<br>67.4+/-5.6<br>NHT >3 months<br>68.3 +/-4.7	RP<br>8.64+/-5.2<br>NHT<br>9.81+/-4.1<br>NHT <3 months<br>10.0 +/-3.9<br>NHT >3 months<br>9.38 +/-4.4	RP<br>1c 37%<br>2 63%<br>NHT<br>1c 37.5%<br>2 62.5<br>n/a in month breakdown	RP<br><=6 44.8%<br>7 31.5<br><=8 23.7<br>NHT<br><=6 41.7%<br>7 34.7<br><=8 23.6<br>NHT <3 months<br><=6 42.6%<br>7 42.6<br><=8 14.9<br>NHT >3 months<br><=6 40%<br>7 20<br><=8 40	RP<br>Low 28.1%<br>Int 27.4%<br>High 44.4%<br>NHT<br>Low 19.4%<br>Int 33.3<br>High 47.2<br>NHT <3 months<br>Low 23.4%<br>Int 36.2<br>High 40.4<br>NHT >3 months<br>Low12%<br>Int 28<br>High 60	RP<br>17.8%<br>NHT<br>19.4%<br>NHT<3 months<br>27.7%<br>NHT>6 months<br>4%	Not available	Not available	Perioperative morbidity of NHT patients undergoing RP appears equivalent to non-NHT patients, with lower positive surgical margin, and PSA recurrence rate
**Narita 2019**	409 patients<br>(RP from diff database)<br>60 – NCHT<br>349 - RP	18 – 22 weeks	NCHT<br>2006 -2016 RP<br>2000 -2014	42.5 months	NCHT<br>65.4 +- 5.3<br>RP<br>66.4 +- 5.9	NCHT<br>26.7+-2.8<br>RP<br>23.4 +-2.3	NCHT<br>1c 31.7%<br>2 36.7%<br>3 31.7%<br>RP<br>1c 36.1%<br>2 30.4%<br>3 31.5%	NCHT<br><=6<br>7<br>>=8<br>RP<br><=6<br>7<br>>=8	Not available	BCR for NCHT<br>2 yr 69.2%<br>5 yr<br>60.1%,<br>BCR rate in the NCHT group was significantly lower than that in the RP alone group (P.021)	Not available	Not available	NCHCT before RP can reduce the risk of BCR in patients with high-risk PCa, particularly if a single high-risk factor is present

**Stewart**<br>**2014**	14575 patients<br>Note - no matching done (generally higher risk group in NHT)	3 months (IQR) 2–4	1987 to 2009	RP 7.7 years<br>(IQR 3.9 – 12.4)<br>NHT 8.3 years (IQR 5–10.8)	RP<br>63 (IQR 57-67)	NHT<br>62 (IQR 56-67)	RP<br>6.0 (4.3-9.0)	NHT<br>7.3 (4.9-13.2)	RP<br>Missing 94<br>T1a/T1b 1.4%<br>T1c 49.9<br>2a 36.5<br>2b 8.5<br>T3/4 3.6	NHT<br>Missing 74<br>T1a/T1b<br>1.1%<br>T1c 37.8<br>2a 35.9<br>2b 11.7<br>T3/4 13.4	RP<br><=6 70.4%<br>7 24.0<br>8-10 5.6	NHT<br><=6 43.6%<br>7 37.9<br>8-1018.5	RP<br>Missing 223<br>Low 47.3%<br>Int 33.4<br>High 19.4	NHT<br>Missing 129<br>Low 23.3<br>Int 36.7<br>High 40	Not available	15 years<br>93% RP<br>84% NHT	RP<br>2.1% death<br>NHT<br>4.5% death	Gleason system appears feasible among hormonally pre-treated prostatectomy specimens and shows continued prognostication for systemic progression
**Tosco**<br>**2017**	Retrospective multi-institutional<br>Propensity matching<br>1573 patients	Not available	1985 to 2015	56 months (interquartile range 29–88)<br>57 – RP<br>54 – NHT	RP<br>66 (61-70)	NHT<br>67 62-71)	RP<br>14 (7-28)	NHT<br>11 (7-25)	RP<br><3 30.9%<br>3 60.9<br>4 2.3<br>Missing 6	NHT<br><3 41.9%<br>3 50.1<br>4 1.5<br>Missing 6.5	RP<br><7 24.2%<br>7 33.9%<br>8-10 40%<br>Missing 2%	NHT<br><7 28%<br>7 27.5%<br>8-10 42.2%<br>Missing 2.2%	Not available		Not available	Not available	Beneficial effect NHT added to RP on PCRD (HR) 0.5; 95% CI 0.32–0.80; *P*=0.0014	NHT before surgery significantly decreased PCRD

GG = grade group; cT = clinical staging; PFS = progression-free survival; CSS = cancer-specific survival; OS = overall survival; NHT = neoadjuvant hormone therapy; RP = radical prostatectomy; HR = hazard ratio; RM = positive resection margin; BCR = biochemical recurrence; Ca = cancer; PCRD = prostate cancer related death; NCHT = neoadjuvant chemohormonal therapy; BRFreeS = biochemical recurrence-free survival; EMP = estramustine phosphate; CHT = chemohormonal therapy; PSA = prostate-specific antigen; IQR = interquartile range; nADT = neoadjuvant hormone therapy

Table S6 – Risk of bias of non-randomised studies comparing NHT and immediate RP

	Study	Selection	Comparability	Outcome
**Kim 2018**	Effect of Neoadjuvant Hormone Therapy on Resection Margin and Survival Prognoses in Locally Advanced Prostate Cancer after Prostatectomy Using Propensity-Score Matching.	★★★	★★	★
**McClintock 2019**	Neoadjuvant Androgen Deprivation Therapy Prior to Radical Prostatectomy: Recent Trends in Utilization and Association with Postoperative Surgical Margin Status.	★★★	★★	★
**Tosco 2017**	The survival impact of neoadjuvant hormonal therapy before radical prostatectomy for treatment of high-risk prostate cancer.	★★★	★	★
**Narita 2019**	Radical Prostatectomy with and without Neoadjuvant chemohormonal pretreatment for High-Risk Localized Prostate Cancer: A Comparative Propensity Score Matched Analysis.	★★	★★	★★
**Fujita 2017**	Overall survival of high-risk prostate cancer patients who received neoadjuvant chemohormonal therapy followed by radical prostatectomy at a single institution.	★★★	★★	★★

**Naiki 2012**	Neoadjuvant hormonal therapy is a feasible option in laparoscopic radical prostatectomy.	★★★	★	★★★
**Stewart 2014**	Gleason grading after neoadjuvant hormonal therapy retains prognostic value for systemic progression following radical prostatectomy.	★★★	★★	★★★
**Hsu 2007**	Comparing results after surgery in patients with clinical unilateral T3a prostate cancer treated with or without neoadjuvant androgen-deprivation therapy.	★★★	★	★★
